# Investigation of *Gynura segetum* root extract (GSrE) induced hepatotoxicity based on metabolomic signatures and microbial community profiling in rats

**DOI:** 10.3389/fmicb.2022.947757

**Published:** 2022-08-09

**Authors:** Xinyi Gu, Shuwei Li, Mengna Lu, Ying Li, Qixue Wang, Long Chen, Yiqun Jia, Shan Cao, Ting Zhang, Mingmei Zhou, Xiaojun Gou

**Affiliations:** ^1^Institute of Interdisciplinary Integrative Medicine Research, Shanghai University of Traditional Chinese Medicine, Shanghai, China; ^2^School of Pharmacy, Shanghai University of Traditional Chinese Medicine, Shanghai, China; ^3^Experiment Center of Science and Technology, Shanghai University of Traditional Chinese Medicine, Shanghai, China; ^4^Central Laboratory, Baoshan District Hospital of Integrated Traditional Chinese and Western Medicine of Shanghai, Shanghai, China

**Keywords:** *Gynura segetum* (Lour.) Merr., hepatotoxicity, metabolomics, gut microbiota, correlation analysis

## Abstract

In recent years, many reports focus on the hepatotoxicity of *Gynura segetum* root extract (GSrE), but the interaction between GSrE and the gut microbiota is still unclear. This study investigated the mechanism of GSrE-induced hepatotoxicity of different doses and exposure durations by combining metabolomics and gut microbiota analysis. SD rats were divided into 3 groups: blank, low-dose (7.5 g/kg), and high-dose (15 g/kg) groups. Urine and feces samples were collected on day 0, day 10, and day 21. Metabolomics based on gas chromatography-mass spectrometry (GC-MS) was carried out to identify metabolites and metabolic pathways. 16S rDNA gene sequencing was applied to investigate the composition of gut microbiota before and after GSrE-induced hepatotoxicity. Finally, a correlation analysis of metabolites and gut microbiota was performed. Differential metabolites in urine and feces involved amino acids, carbohydrates, lipids, organic acids, and short chain fatty acids. Among them, L-valine, L-proline, DL-arabinose, pentanoic acid, D-allose, and D-glucose in urine and D-lactic acid and glycerol in fecal metabolites depended on the exposure of time and dose. In addition, 16S rDNA sequencing analysis revealed that GSrE-induced hepatotoxicity significantly altered the composition of gut microbiota, namely, f_*Muribaculaceae*_Unclassified, *Lactobacillus, Bacteroides, Lachnospiraceae*_NK4A136_group, f_*Ruminococcaceae*_Unclassified, *Prevotellaceae*_Ga6A1_group, and *Escherichia*-*Shigella*. The correlation analysis between gut microbiota and differential metabolites showed the crosstalk between the gut microbiota and metabolism in host involving energy, lipid, and amino acid metabolisms. In summary, our findings revealed that peripheral metabolism and gut microbiota disorders were time- and dose-related and the correlation between gut microbiota and metabolites in GSrE-induced hepatotoxicity.

## Introduction

*Gynura segetum* (Lour.) Merr. root extract (GSrE) (Tusanqi or Jusanqi), a traditional Chinese medicine herb, has the effect of promoting circulation, relieving pain, and removing stasis and has been widely used in traumatic injury and Chinese folk medicine according to Zhang et al. (2022b). The plant is also known for its treatment of cancer, inflammation, diabetes, hypertension, and skin afflictions (Seow et al., 2014). The plant is also known for its treatment of cancer, inflammation, diabetes, hypertension, and skin afflictions (Seow et al., 2014), though there are no clinical randomized controlled trials. GSrE is often misused as *Panax notoginseng*, a famous Chinese herbal medicine, and causes hepatotoxicity. Among the major compounds found in GSrE, pyrrolizidine alkaloids (PAs) can induce severe toxic reactions, which is one of the main reasons for the hepatotoxicity of GSrE. In recent years, many reports focused on the hepatotoxicity caused by GSrE (Zhu et al., 2021; Zhang et al., 2022b). PA is the most hepatotoxic natural compound (Stegelmeier et al., 1999), and most PAs are metabolically activated by cytochrome P450 (CYP450) and then form dehydropyrrolizidine alkaloid (DHPA), a highly active metabolite, which is a chemically reactive electrophilic metabolite with an extremely short half-life. DHPAs will interact with cellular macromolecules quickly, form 2, 3-dihydro-1H-pyrroleazine protein (pyrrole protein) adducts, and then exacerbate liver injury such as hepatic sinusoidal obstruction syndrome (Yang et al., 2016). Furthermore, while saturated PAs are harmless, 1,2-unsaturated PAs are converted into toxic PA radicals by removing the double bond between C1 and C2 (Teschke et al., 2021). This transformation occurs in the endoplasmic reticulum, which corresponds to the microsomal fraction of hepatocytes. PA radicals bind to hepatocytes or blood proteins and produce pyrrolizidine adducts, which are considered as biomarkers of hepatotoxicity in clinical diagnosis (Teschke et al., 2021). Herbal medicines containing PAs have attracted the attention of countries such as Germany, Ghana, and North America, suggesting that regulators conduct stricter quality control testing of PAs to minimize consumer exposure to these toxic compounds (Roeder et al., 2015; Letsyo et al., 2017a,b). Another study found that, after successive administration of different doses of *Gynura segetum* decoction to rats, *Gynura segetum* led to ingravescence of hepatotoxicity with a dose-dependent relationship. However, except for the research above, the toxicological mechanism of *Gynura segetum* is still unclear.

Metabolomics, a part of systems biology, is used to clarify the small molecule metabolite profile of organisms and has applications in toxicology, clinical research diagnosis, etc. The effect of GSrE on endogenous metabolites in the body is still unknown and only a few studies reported the effect of GsrE on endogenous metabolites (Qiu et al., 2018). Therefore, metabolomic methods can elucidate the changes of metabolites in different tissues of the body exposed to GSrE and help to find differential biomarkers.

In addition, gut microbiota has been demonstrated to modulate many extraintestinal organ diseases, such as liver injury. For example, Chen et al. (2015b), found that fatty acids produced by gut microbiota can protect against alcohol-induced liver damage. However, the specific role of gut microbiota on drug-induced liver injury and toxicity remains to be elucidated.

Although a considerable number of studies focused on the hepatotoxicity induced by GSrE, there is still a lack of research on the mechanism of GSrE-induced hepatotoxicity based on gut microbiota. In this study, we first combined urine and fecal metabolomics with 16S rDNA gene sequencing to explore the specific mechanism of GSrE-induced hepatotoxicity from a time- and dose- related perspective.

## Materials and methods

### Reagents and instruments

*Gynura segetum* (purchased from Bozhou, Anhui, China) was identified as the root of *Gynura segetum* (Lour.) Merr. by Professor Yajun Cui (School of Pharmacy, Shanghai University of Traditional Chinese Medicine, Shanghai, China.). The plant was stored in the laboratory of the Shanghai University of Traditional Chinese Medicine. *Gynura segetum* roots were chopped and suspended in distilled water for 2 h. The mixture was then boiled for 1.5 h and filtered. The entire extraction procedure was repeated one time, and the extracts were merged and equilibrated to 1.5 g/mL.

Urease (Lot: SLBB0100V) was purchased from Sigma-Aldrich; methanol (AR) was purchased from Sinopharm Chemical Reagent Co., Ltd; nonadecanoic acid (Lot: E0810030) was purchased from ANPEL Instrument Co. Ltd; 2-chloro-phenylalanine was purchased from Aladdin; methoxyamine hydrochloride (Lot: BCBP2843V) was purchased from Sigma-Aldrich; pyridine (AR, 10018118) was purchased from Sinopharm Chemical Reagent Co., Ltd; BSTFA with 1% TMCS (Lot: 62894) was purchased from REGIS Technologies, Inc. The instruments were: Gas chromatography-mass spectrometry (GC-MS) (Agilent 7890A, 7000B QQQ-MS detector); 1730R centrifuge (Gene Company Limited); Tissue Lyser II homogenizer (Qiagen).

### Animal treatment

A total of 48 male SD rats (160–200 g) were purchased from Shanghai SLAC Laboratory Animal Co. Ltd. (approval number: SCXK 2017-0005) and fed at the Laboratory Animal Center of Shanghai University of Traditional Chinese Medicine, at 22–25°C room temperature and 45%–70% relative humidity. Animal welfare is strictly implemented following “The Guide for Care and Use of Laboratory Animals” and the ethics and regulations of Shanghai University of Traditional Chinese Medicine.

After 1 week of adaptive feeding, animals were randomly divided into 3 groups: the blank group (B, *n* = 16); the low dose group (L, *n* = 16); and the high dose group (H, *n* = 16). The high-dose group and the low-dose group were given 15 and 7.5 g/(kg^*^d) of GSrE water decoction for 21 days, respectively (Zhang et al., 2022a). The clinical equivalent dose of 7.5 g/kg in rats is 500 g in humans (Song et al., 2011). The rats in the blank group were given an equal volume of purified water. On day 10, half of the animals in each group were sacrificed for liver injury assessment, specifically, there were 16 rats in each group on day 0, and there were 8 rats in each group on days 10 and 21. While some rats died due to hepatotoxicity induced by high-dose GSrE administration, only 4 rats survived on day 21 in the high-dose group.

### Sample collection and preparation

Urine and fecal samples of each rat were collected on days 0, 10, and 21 according to the degree of hepatotoxicity with metabolic cage and stored at −80°C for further analysis in accordance with techniques found in previous studies by Gou et al. (2017) and Qiu et al. (2018). Animals were euthanized at the end of the trial.

Thaw urine samples at room temperature and centrifuged for 10 min (12,000 rpm, 4°C). A supernatant of 100 μL was collected and 50 μL of urease water solution (4 mg/ml) was added and reacted at 37°C for 90 min. Then, 300 μL of methanol and 30 μL of internal standard (0.2 mg/ml nonadecanoic acid and 2-chloro-phenylalanine solution) were added, centrifuged for 10 min (10,000 rpm, 4°C), and finally, 200 μL of the supernatant was collected.

Then, 500 μL of purified water was added to the centrifuge tube with 100 mg of the fecal sample. The samples were homogenized for 5 min, centrifuged for 10 min (13,000 rpm, 4°C), and 400 μL of the supernatant was collected as the first extract. Later, 500 μL of methanol was added to the above fecal sediment and the mixture was homogenized and centrifuged again. Again, 400 μL of the supernatant was obtained and mixed with the first extract. The mixture was centrifuged for 10 min (13,000 rpm, 4°C) and 200 μL of the supernatant was collected as the second extract. Finally, 30 μL of internal standard was added to the supernatant.

Freeze-drying was applied to the supernatant of urine and fecal samples above and reconstituted with 50 μL of methoxyamine hydrochloride pyridine solution (20 mg/mL). Samples were subjected to methoxylation reaction at 37°C for 90 min. After the reaction, 30 μL of BSTFA with 1% TMCS was added and the silylation reaction was carried out at 70°C for 60 min. After being placed at room temperature for 60 min, the samples were analyzed on the GC-MS.

### Metabolomic analysis

The GC-MS column was Agilent VF-WAXms (30 m × 0.25 mm × 0.25 μm). The GC parameters are as follows: high-purity helium (purity: 99.9996%) was the carrier gas, the injection port temperature was 280°C with splitless injection (1.0 μL injection volume), and the flow rate was 1.0 mL/min. The initial temperature was 80°C and lasted for 2 min. Then, the temperature was raised to 300°C at a rate of 10°C/min and kept for 6 min. The MS parameters are as follows: the ion source temperature was 230°C, the quadrupole temperature was 150°C, and the mass spectrometer interface temperature was 280°C. The solvent delay time was 5.65 min, ionization mode was EI, electron impact ionization voltage was 70 Ev, and scan range (m/z) was 50-600.

The raw data were imported into R software (v2.13.2) for data preprocessing. Principal component analysis (PCA) and orthogonal partial least squares-discriminant analysis (OPLS-DA) were performed with SIMCA software (v14.0, Umetrics AB, Umeå, Sweden). Student's *t*-test was applied for statistical significance (*p*-value). Metabolites with variable importance in the project (VIP) > 1 and a *p*-value < 0.05 were considered differential metabolites. The NIST database was applied to perform identification of the significantly differential metabolites by their *m*/*z*, mass spectra, and retention time. Kyoto Encyclopedia of Genes and Genomes (KEGG, http://www.genome.jp/kegg/) and Human Metabolome Database (HMDB, http://www.hmdb.ca/) database were used to annotate the metabolites. MetaboAnalyst 5.0 (https://www.metaboanalyst.ca/) database was used for pathway analysis.

### Microbial community profiling

Total genome DNA from feces was extracted by Soil DNA Kit (Omega Bio-Tek, Norcross, GA, United States) and DNA concentration was monitored by Qubit3.0 Fluorometer.

The microbial DNA regions V3–V4 of the bacterial 16S rDNA gene were amplified with forward and reverse primers containing “CCTACGGRRBGCASCAGKVRVGAAT” and “GGACTACNVGGGTWTCTAATCC”. Meanwhile, indexed adapters were added to the ends of the 16S rDNA amplicons to generate indexed libraries for downstream NGS sequencing on Illumina Miseq. PCR reactions were performed in triplicate with a 25-μL mixture containing 2.5 μL TransStart Buffer, 2 μL dNTPs, 1 μL each primer, and 20 ng template DNA.

PE250/pe300 double-ended sequencing was carried out according to the operation method of Illumina MISeq (Illumina, San Diego, CA, USA), and MISeq Control Software was used to read the sequence information.

After quality filter and purifying chimeric sequences, VSEARCH (1.9.6) was applied to perform an operational taxonomic unit (OTU) clustering of sequences based on 97% similarity.

Then, the Bayesian algorithm of the RDP classifier (Ribosomal Database Program) was used for the taxonomic analysis of representative sequences of OTUs and for counting the community composition of each sample at different taxonomic levels. Alpha diversity index (Chao1 index and Shannon index) and beta diversity (PCA) was performed based on OTU analysis results.

### Correlation analysis and statistical analysis

The statistical analysis was carried out by SPSS software, version 21.0 (SPSS; IBM, Armonk, NY, USA). Spearman's correlation coefficient was used to assess the correlation between gut microbiota at the genus level and urine metabolites or fecal metabolites, and the results were shown with heatmaps. A one-way ANOVA and the two-tailed Student's *t*-test were applied for significant differences analysis. All the data were presented as mean ± SD. A *p*-value of < 0.05 was considered statistical significance.

## Results

First, after the administration of GSrE with low and high doses, the levels of serum ALT and AST were significantly higher than the blank group both on days 10 and 21 (Supplementary Table S1). If liver cells are damaged and destroyed, AST and ALT enzymes in liver cells will enter the blood. When both the enzymes are elevated at the same time, it indicates liver injury. Nevertheless, the results of liver histopathology showed that the liver tissue of the rats in the blank group had a clear structure, a neat arrangement, and a complete hepatocyte morphology (Supplementary Figure S). However, after GSrE administration, the arrangement of hepatocytes was disordered, namely, disordered arrangement, necrosis, and vacuolar degeneration of hepatocytes, unclear cell boundary, and sinusoidal hemorrhage (Supplementary Figure S1). These findings mean that the GSrE-induced liver injury model was successful.

### Metabolic pattern in each group of urine and fecal samples

#### PCA results of urine and fecal samples

**Figure 1:**
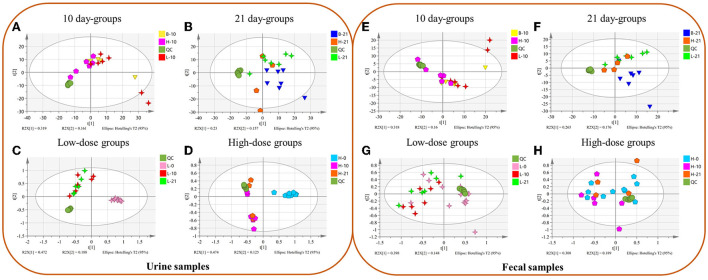
The Principal Component Analysis (PCA) between different groups of urine **(A–D)** and fecal **(E–H)** samples. **(A**,**E**) Different doses of 10 day-groups; **(B,F)** different doses of 21 day-groups; **(C,G)** 0, 10, 21 days of low-dose groups; **(D,H)**, 0, 10, 21 days of high-dose groups. (*n* = 4–8).

In urine metabolomics, PCA results of the 10-day groups and the 21-day groups did not reveal separation (Figures 1A,B). The results in the low-dose groups and high-dose groups showed a separation between day 0 and days 10 and 21 (Figures 1C,D). In fecal metabolomics, all the PCA results did not show an apparent separation (Figures 1E–H).

#### OPLS-DA results and differential metabolites of urine metabolomics

**Figure 2:**
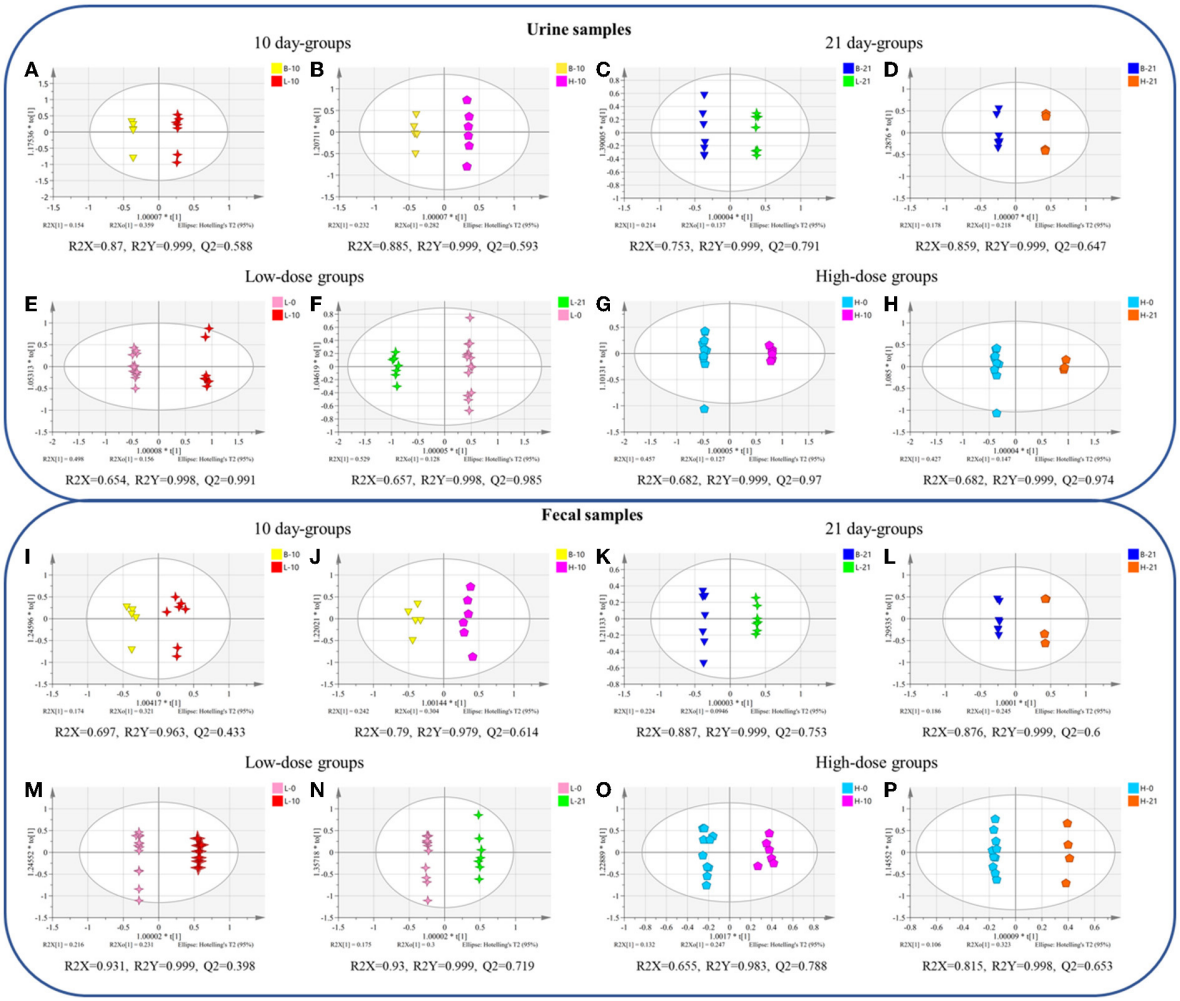
The orthogonal partial least squares-discriminant analysis (OPLS-DA) between different groups of urine **(A–H)** and fecal **(I–P)** samples. **(A,I)** blank group vs. low-dose group on the 10th day; **(B,J)** blank group vs. high-dose group on the 10th day; **(C,K)** blank group vs. low-dose group on the 21st day; **(D,L)** blank group vs. high-dose group on the 21st day; **(E,M)** 0-day vs. 10-day of low-dose groups; **(F,N)** 0-day vs. 21-day of low-dose groups; **(G,O)** 0-day vs. 10-day of high-dose groups; **(H,P)** 0-day vs. 21-day of high-dose groups. (*n* = 4–8).

The OPLS-DA plot exhibited a clear separation between the blank groups and the low-dose or high-dose GSrE administration groups after day 10 and day 21 (Figures 2A–D). On day 10, the endogenous differential metabolites from the low- and high-dose groups as compared with those from the blank group were 13 and 20, respectively, of which 5 metabolites were common, involving amino acids and carbohydrates (Supplementary Table S2). After a low dose of GSrE administration for 10 days, the level of glycerol accumulated significantly, which is the substrate for gluconeogenesis and could cause hepatic oxidative stress (Abugomaa and Elbadawy, 2020). Pertaining to the different dose groups, the number of differential metabolites between B-10 and H-10 was more than that between B-10 and L-10, which suggests that GSrE-induced hepatotoxicity may be dose related. For example, D-lactic acid was differentially expressed only at high doses of administration, which is commonly seen in the models of hepatotoxicity according to a study by Geng et al. (2020). The content of pyrimidine decreased significantly in the GSrE high-dose group, suggesting the disorder of pyrimidine and purine metabolism, which is related to liver lipid accumulation Le et al. (2013). Low-dose and high-dose administration each modulated some short chain fatty acids (SCFAs), namely, acetic acid and pentanoic acid in the low-dose groups and propanoic acid and butanoic acid in the high-dose groups. After administration of low- and high-dose GSrE for 21 days, the endogenous differential metabolites were 20 and 11, respectively, compared with the blank group, of which 8 metabolites were common, including amino acids, SCFAs, and carbohydrates (Supplementary Table S2). The level of D-lactic acid changed significantly in the low-dose group after administration for 21 days. Phosphoric acid and glycerol, which are common in liver injury (Lin et al., 2021), were significantly changed in the high-dose group, whereas 5-methyluridine in the low-dose group and uridine in the high-dose group were significantly changed, both of which are common metabolites in hepatotoxicity. In conclusion, in terms of dose-dependence, the high-dose group modulated more metabolites on day 10, although the high-dose group modulated fewer metabolites than the low-dose group on day 21, which may due to the smaller number of the samples in the 21-day high-dose group. Some differential metabolites occurred only after high dose administration, such as D-Lactic acid, phosphoric acid, pyrimidine, and uridine, which may depend on the dose.

In terms of time-related, the OPLS-DA scores plot also showed clear discrimination between the groups on day 0, and the groups on day 10 or day 21 with low-dose or high-dose GSrE administration (Figures 2E–H). In the low-dose groups, compared with the day 0, 34 and 36 metabolites were regulated on days 10 and 21, respectively, of which 33 metabolites were shared, including amino acids, carbohydrates, SCFAs, and organic acids. (Supplementary Table S3). L-methionine levels decreased after 21 days of low-dose GSrE administration and showed time-related features, although the study found that the high-methionine diet might increase ethanol-induced hepatotoxicity and oxidative stress (Yalçinkaya S, 2007). At the same time, the content of butanoic acid was significantly enriched. On the other hand, in the high-dose groups, 34 and 32 metabolites were regulated on day 10 and day 21, respectively, of which 31 metabolites were shared, similar to the low-dose groups (Supplementary Table S3). Overall, in the low-dose groups, there were more differential metabolites on day 21 than on day 10, which may be related to time.

**Figure 3:**
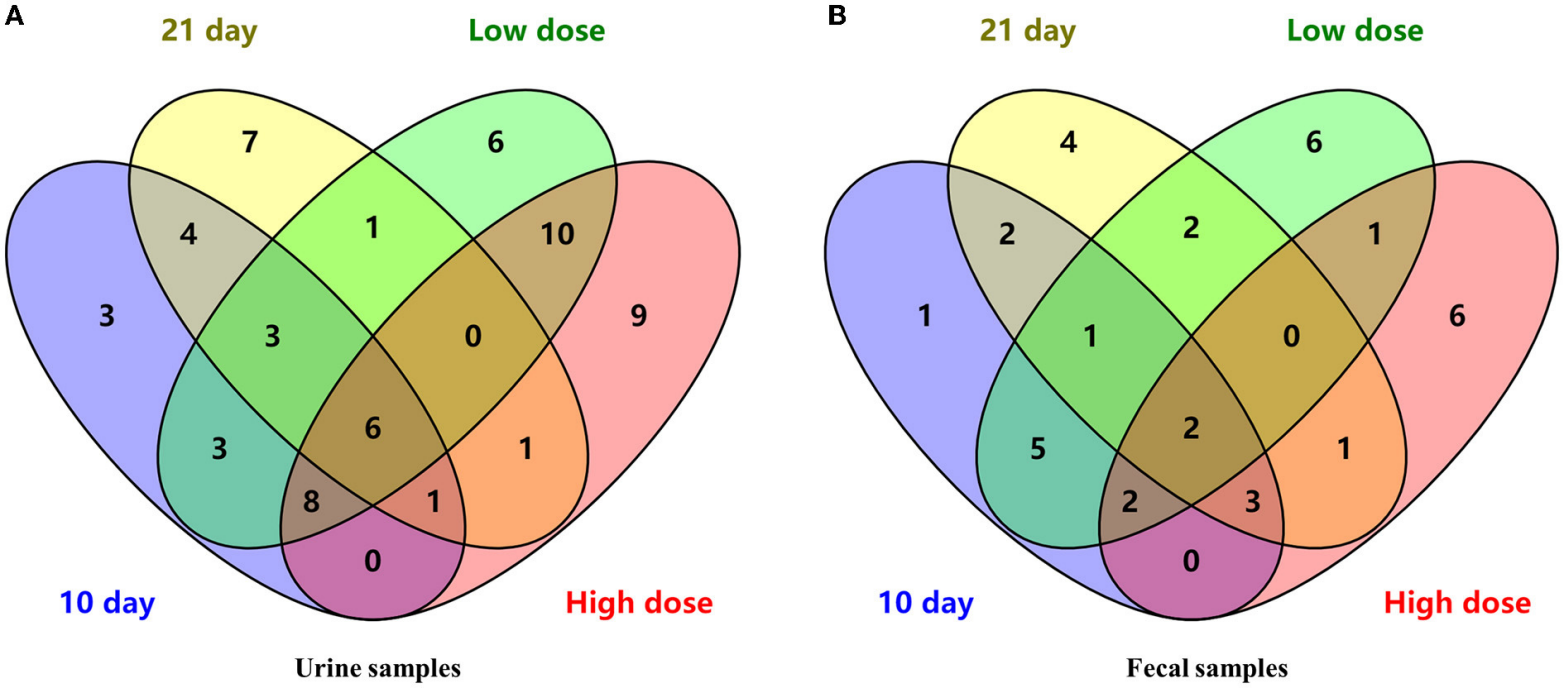
The Venn diagram of differential metabolites in urine **(A)** and fecal **(B)** samples.

Combining the dose- and time-related results, a Venn diagram was applied (Figure 3A) and 6 differential metabolites were obtained, namely, L-valine, L-proline, DL-arabinose, pentanoic acid, D-allose, and D-glucose.

#### OPLS-DA results and differential metabolites of fecal metabolomics

In terms of the dose-related, between the blank and low- or high-dose groups on day 10 and day 21, the OPLS-DA scores plot all showed clear separation (Figures 2I–L). On day 10, compared with the blank group, the low-dose and high-dose groups regulated 7 and 13 metabolites, respectively, and 4 metabolites were in common (Supplementary Table S4). Low-dose administration for 10 days reduced the level of butanoic acid in feces. Similar to the urine metabolomics results, the levels of D-lactic acid and glycerol were significantly altered in the high-dose group. On day 21, 10 and 11 metabolites were regulated by the low-dose and high-dose groups, compared to the blank group, respectively, of which 6 metabolites were in common (Supplementary Table S4). Among them, inositol is an essential nutrient source for life and has the effect of reducing hepatic triglycerides and cholesterol accumulation as reported by Pani et al. (2020). Similar to the results of the urine metabolomics, differential metabolites of feces in the 21-day high-dose group also included phosphoric acid and glycerol. In conclusion, the high-dose groups modulated more metabolites than the low-dose groups on days 10 and 21, indicating dose-related hepatotoxicity.

Time- related OPLS-DA scores plot also exhibited significant separation between the groups on day 0 and the groups on day 10 or 21 with low-dose or high-dose GSrE administration (Figures 2M–P). In the low-dose groups, 15 and 12 metabolites were regulated, respectively, on the 10-day and 21-day groups compared with the 0-day group, of which 8 metabolites were altered (Supplementary Table S5). Especially butanoic acid was significantly enriched on day 10 with the low-dose administration. In addition, in the high-dose groups, 9 and 8 metabolites were regulated on days 10 and 21, respectively, and 2 metabolites were in common, namely, glycerol and isoquinoline (Supplementary Table S5). On day 10 of high-dose administration, inositol and D-lactic acid were significantly changed. On day 21, the content of some carbohydrates decreased significantly, which depended on time to some extent.

Overall, a Venn diagram was applied (Figure 3B) and 2 differential metabolites were both dose- and time- related, namely, D-lactic acid and glycerol.

#### Metabolic pathway analysis of urine metabolites

**Figure 4:**
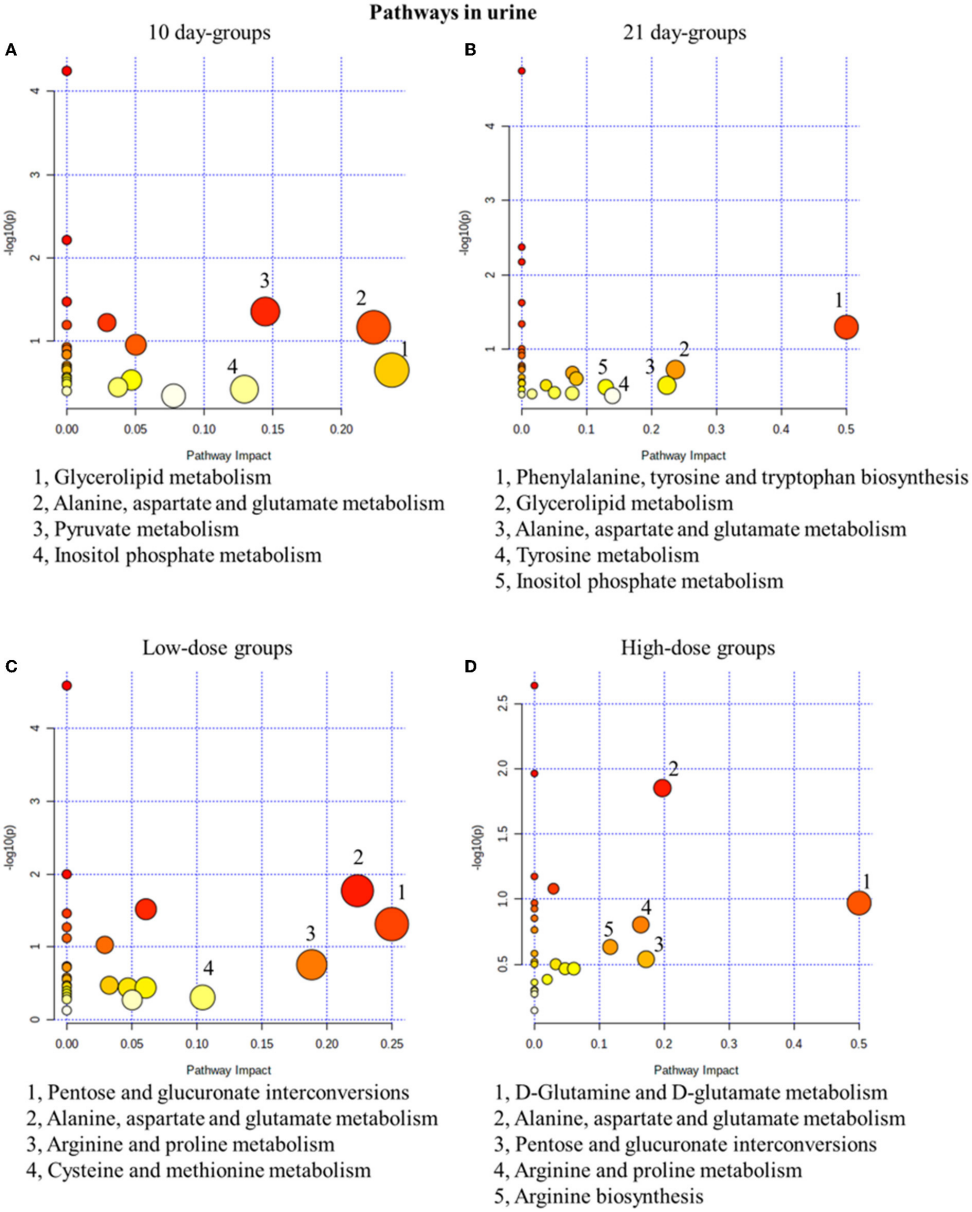
Metabolic pathway analysis of urine samples with different groups. **(A)** pathways of low-dose and high-dose groups on the 10th day. **(B)** pathways of low-dose and high-dose groups on the 21th day. **(C)** pathways on the 10th day and 21st day of low-dose groups. **(D)** pathways on the 10th day and 21st day of high-dose groups.

The pathway analysis results were screened with an impact >0.1, and the results showed that in the 10-day groups and the 21-day groups, 4 and 5 metabolic pathways were defined as disturbed, respectively (Figure 4). Glycerolipid metabolism, alanine, aspartate and glutamate metabolisms, and inositol phosphate metabolism were all present in the 10-day groups and the 21-day groups. In addition, low-dose administration regulated 4 pathways in the 10-day and 210-day groups and 5 pathways in high-dose administration groups (Figure 4). Pentose and glucuronate interconversions, alanine, aspartate and glutamate metabolisms, and arginine and proline metabolisms were the common pathways in the low-dose and high-dose groups. Alanine, aspartate, and glutamate metabolisms were both time- and dose-related pathways, which have been proved to be closely related to the mechanism of drug toxicity according to Hu et al. (2021a).

#### Metabolic pathway analysis of fecal metabolites

**Figure 5:**
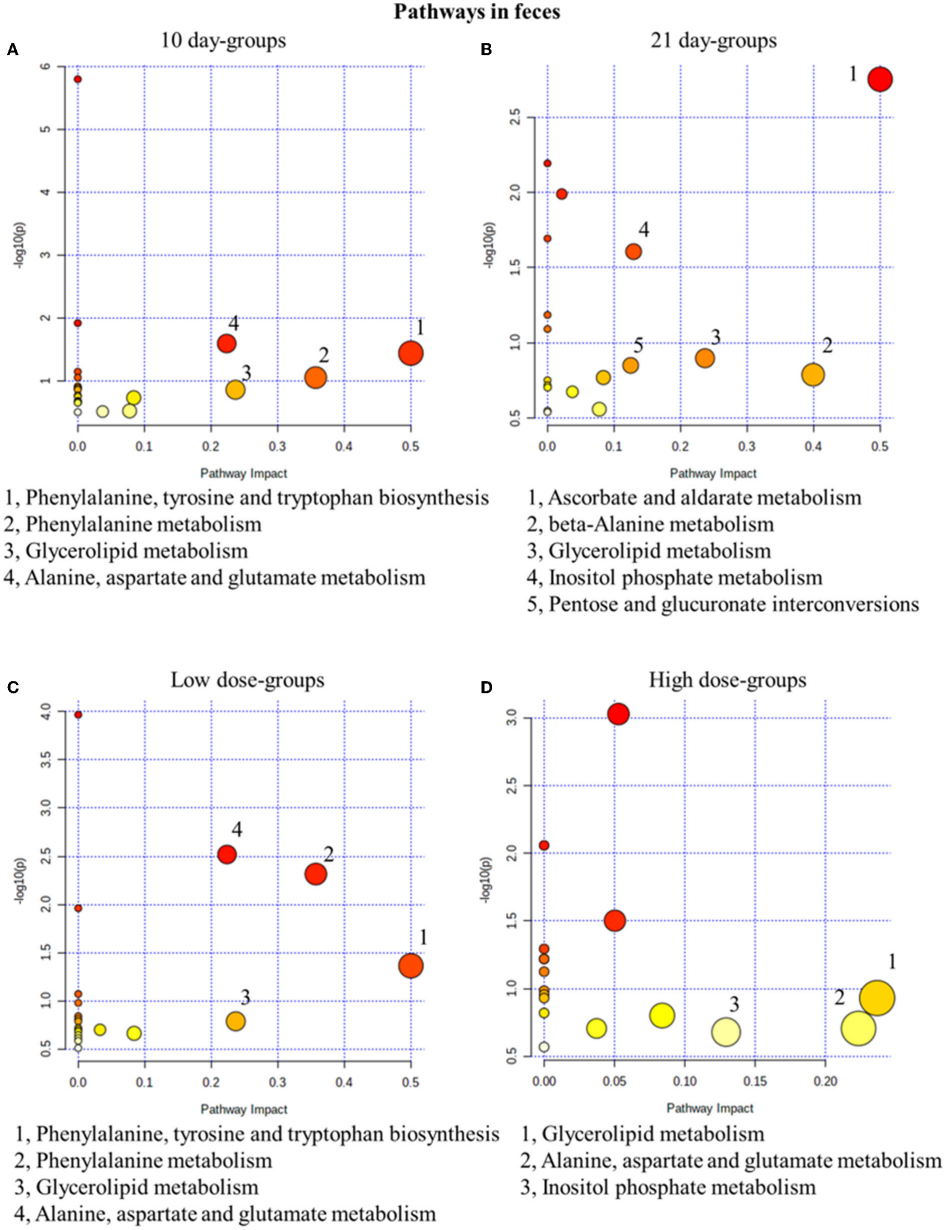
Metabolic pathway analysis of fecal samples with different groups. **(A)** Pathways of low-dose and high-dose groups on the 10th day. **(B)** Pathways of low-dose and high-dose groups on the 21th day. **(C)** Pathways on the 10th day and 21st day of low-dose groups. **(D)** Pathways on the 10th day and 21st day of high-dose groups.

Similar to the results in urine results, in the 10-day groups and the 21-day groups, 4 and 5 metabolic pathways were significantly changed, respectively (Figure 5). In fact, 4 and 3 pathways were disturbed in the low- and high-dose groups (Figure 5). In conclusion, glycerolipid metabolism was the most related pathway, which is both time- and dose-related. In our fecal metabolomics, the level of glycerol was significantly increased after GSrE administration, and glycerol is one of the main classes of lipid. Studies found that disturbed lipid metabolism is common in liver injury models (Liang et al., 2016; Li et al., 2020b).

### Gut microbiota composition analysis

#### The diversity of the gut microbiota

**Figure 6:**
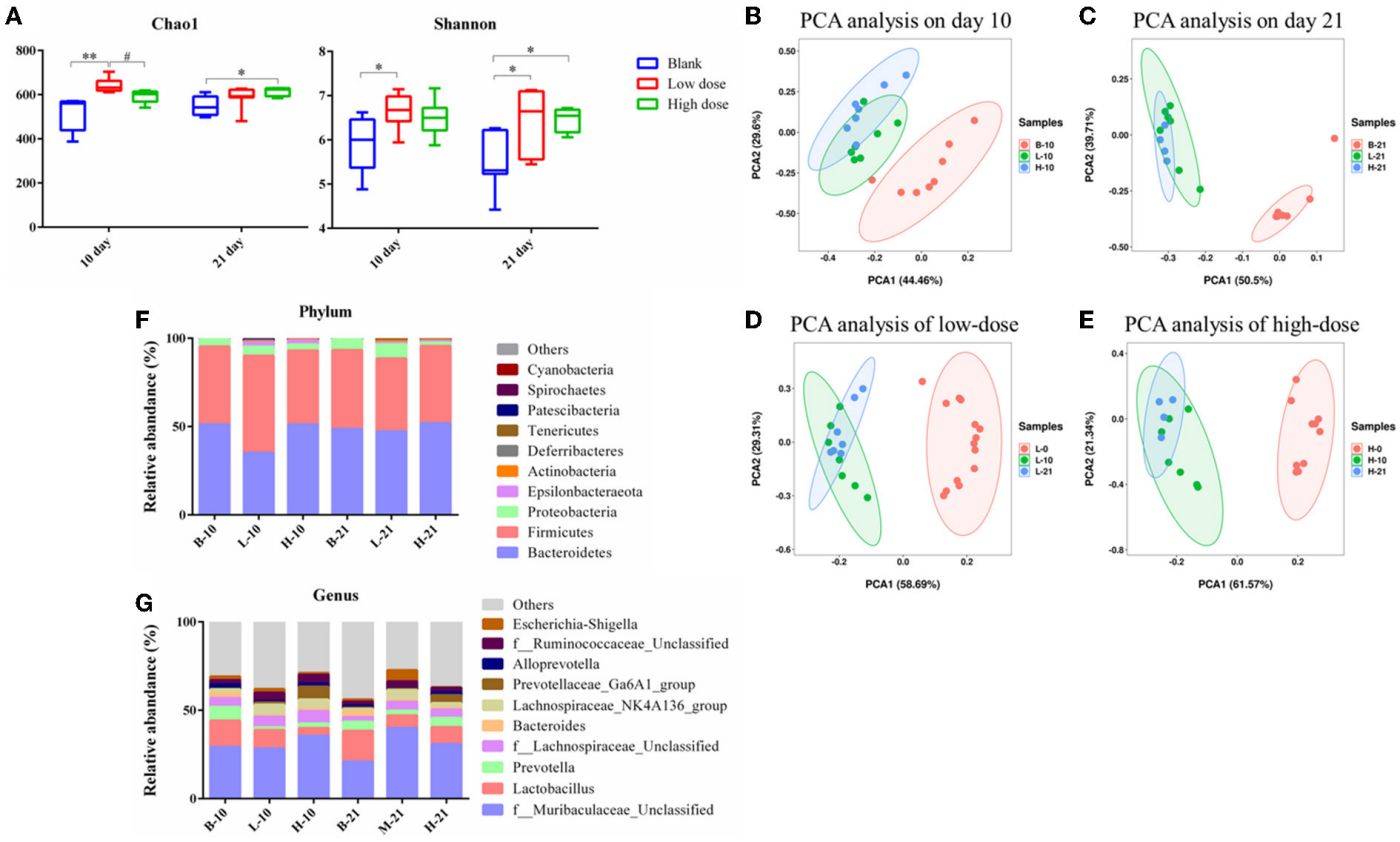
Gut microbiota analysis of each group. **(A)** Alpha diversity analysis. **(B–E)** PCA analysis on the 10th day groups **(B)**, 21st day groups **(C)**, low-dose groups **(D)**, and high-dose groups **(E)**. **(F)** Relative abundance of significantly altered taxa at the phylum level. **(G)** Relative abundance of significantly altered taxa at the genus level. * *P* < 0.05, compared with the blank groups; ** *P* < 0.01, compared with the blank groups; # *P* < 0.05, compared with the low dose groups, *n* = 4–8.

The Chao1 index and the Shannon index were determined for the alpha diversity analysis. As seen in Figure 6A, after low-dose GSrE administration induced hepatotoxicity for 10 days, the value of Chao1 and Shannon significantly increased, and only the value of Shannon improved remarkably on the 21-day groups. In the high-dose groups, except that the value of Chao1 increased significantly on the 21-day, the value of Chao1 and Shannon were all decreased (Figure 6A). These indicated that low-dose GSrE administration increased the diversity of gut microbiota, while high-dose administration decreased the diversity. At the same time, the same-dose groups had similar diversity on days 10 and 21.

The PCA score was used for the beta diversity between different time points and different doses. As seen in Figures 6B,C, on day 10, the low-dose and high-dose groups were separated from the blank group, and the trend was more obvious on day 21. Between the low- and high-dose groups, the separation was not obvious both on days 10 and 21. For the 10-day and 21-day low-dose groups, gut microbiota composition was separated from day 0, while the composition was overlapped between the 10-day and 21-day groups (Figure 6D). The segregation trend of gut microbiota at the three time points of high-dose administration was similar to that of low-dose administration (Figure 6E).

#### Analysis of the gut microbiota composition at phylum and genus level

**Figure 7:**
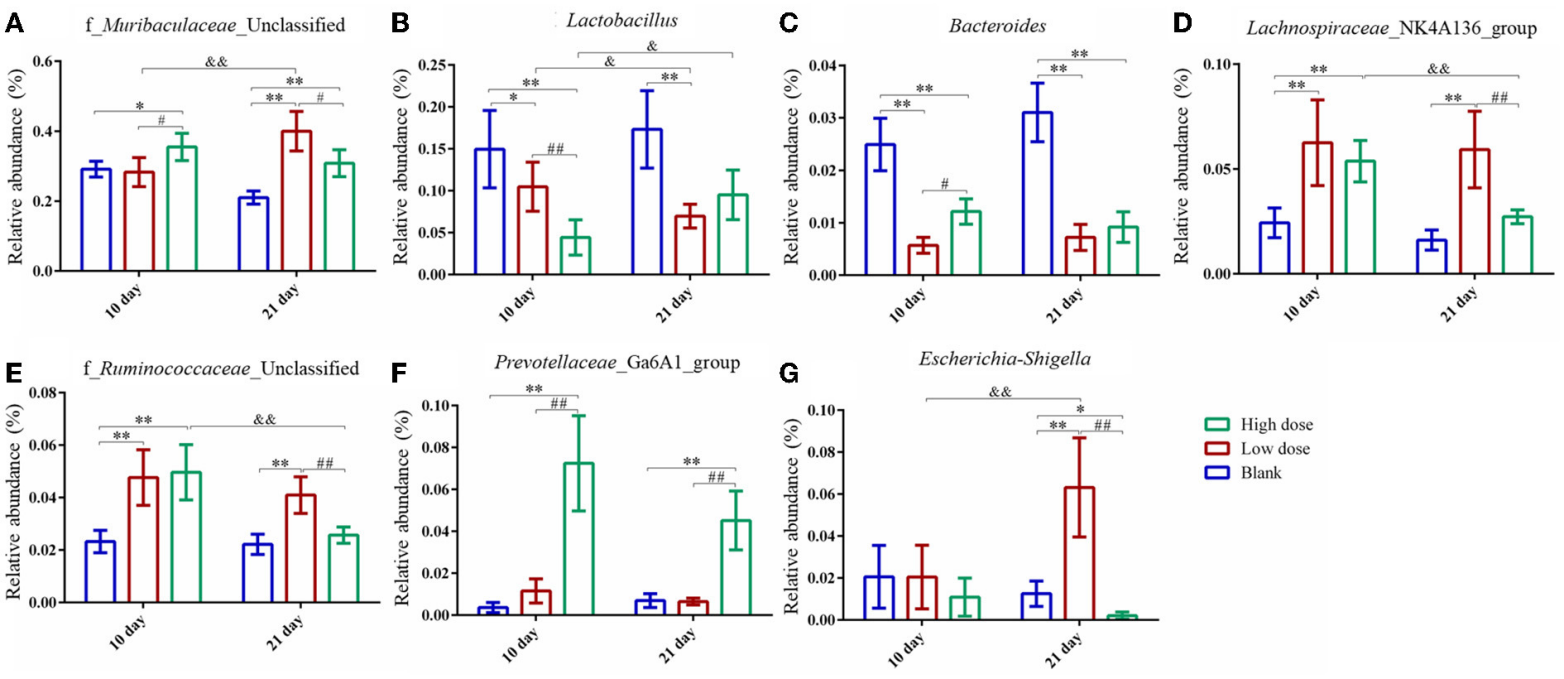
Species with significantly relatively high abundance and significant differences at genus level. **(A)** f_*Muribaculaceae*_Unclassified; **(B)** *Lactobacillus*; **(C)** *Bacteroides*; **(D)** *Lachnospiraceae*_NK4A136_group; **(E)** f_*Ruminococcaceae*_Unclassified; **(F)** *Prevotellaceae*_Ga6A1_group; **(G)** *Escherichia-Shigella*. * *P* < 0.05, ** *P* < 0.01, compared with the blank groups; # *P* < 0.05, ## *P* < 0.01, compared with the low dose groups; & *P* < 0.05, && *P* < 0.01, comparison between groups at the same dose, *n* = 4–8.

The relative abundance of the gut microbiota with the most significant changes at the phylum level showed that the most dominant gut microbiota in each group were *Bacteroidetes* and *Firmicutes* (Figure 6F). About 90% of the microbes in the intestines belong to *Firmicutes* and *Bacteroidetes* phyla according to Eckburg et al. (2005). At the genus level, the most differential microbiota are seen in Figure 6G. Seven single species with relatively high proportions and significant differences between the groups are counted in Figure 7. Among them, the abundance of f_*Muribaculaceae*_Unclassified, *Lachnospiraceae*_NK4A136_group, *Prevotellaceae*_ Ga6A1_group, f_*Ruminococcaceae*_Unclassified, and *Escherichia*-*Shigella* were enriched after administration of GSrE, while the abundance of *Lactobacillus* and *Bacteroides* were downregulated (Figure 7). The abundance of f_*Muribaculaceae*_Unclassified (Phylum *Bacteroidetes*) was increased after low- and high-dose GSrE administrations and continued to rise after low-dose administration (Figure 7A), which is often seen in the nonalcoholic fatty liver disease and obesity as observed in the studies by Xia et al. (2021); Lan et al. (2022). *Lactobacillus* (Phylum *Firmicutes*) is a probiotic, and its abundance decreased significantly after GSrE administration and with the increase of the dose on day 10 (Figure 7B). In the low-dose groups, the abundance also decreased significantly on day 21 compared with day 10, which shows a time- and dose-dependent relationship. *Bacteroides* (Phylum *Bacteroidetes*), a beneficial bacterium, was significantly decreased after the administration of different doses of GSrE (Figure 7C). The abundance of *Lachnospiraceae*_NK4A136_group (Phylum *Firmicutes*) and f_*Ruminococcaceae*_Unclassified (Phylum *Firmicutes*), both increased after GSrE administration, and with the advancement of time and the increase of dose, the abundance decreased in the high-dose group on day 21 (Figures 7D,E). The study found that these two bacteria belonged to *Lachnospiraceae* and *Ruminococcus* at the family level, which both increased in the hepatotoxicity models proposed by Chen et al. (2015a). *Prevotellaceae*_Ga6A1_group (phylum *Bacteroidetes*) had low abundance in the blank and low-dose groups, and the abundance increased significantly after high-dose administration (Figure 7F). *Escherichia*-*Shigella* (phylum *Proteobacteria*) is a pathogenic bacterium, the proportion of which was higher in the sepsis-related liver injury rats shown in a study by Liang et al. (2022). Our results also showed a significant increase in abundance in the low-dose group on day 21, while among the three groups on day 10, there was no significant difference (Figure 7G).

### Correlation analysis between the urine or fecal metabolomics and gut microbiota analysis

To further understand the correlation of metabolomic characteristics and gut microbiota communities, the covariation relationship between differential urine and fecal metabolites and the differential gut microbiota at the genus level were manifested by heatmaps.

**Figure 8:**
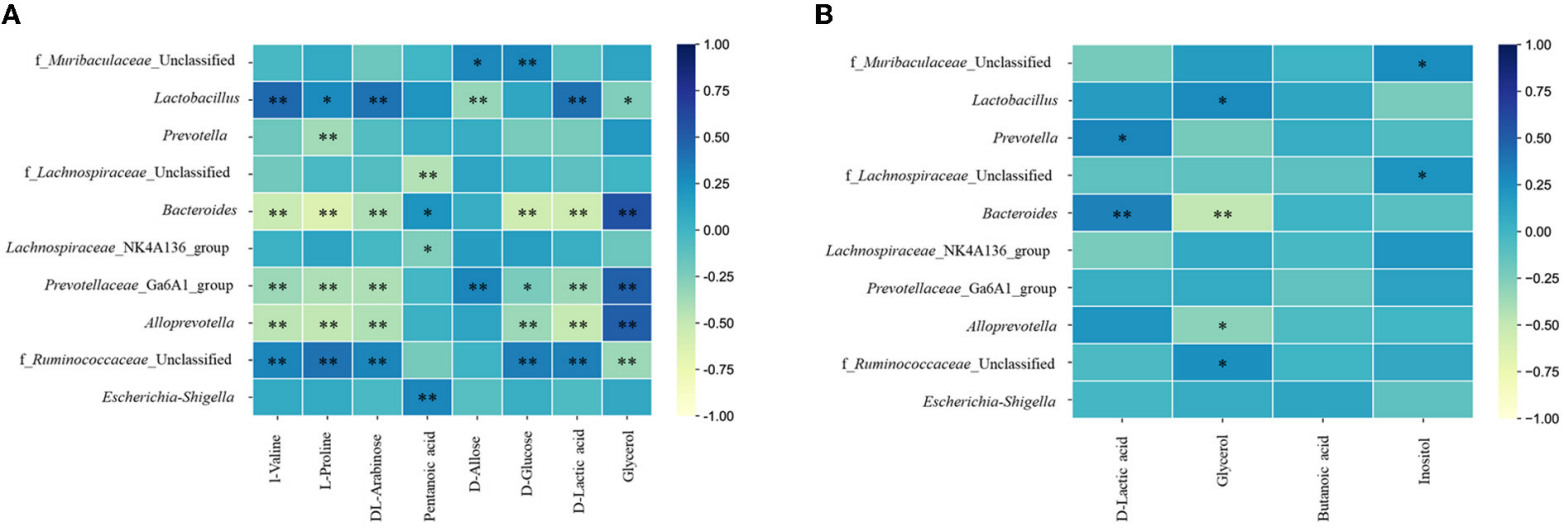
Heatmaps of correlation analysis between top 10 abundance species at genus level and urine **(A)** or fecal **(B)** metabolites. * *P* < 0.05; ** *P* < 0.01.

The urine metabolites had a strong correlation with the top 10 gut microbiotas at the genus level (Figure 8A). In fact, D-allose and D-glucose were positively correlated with f_*Muribaculaceae*_Unclassified. L-valine, L-proline, and DL-arabinose had a significant positive correlation with *Lactobacillus* and f_*Ruminococcaceae*_Unclassified and a negative correlation with *Bacteroides, Prevotellaceae*_Ga6A1_group, and *Alloprevotella*. Pentanoic acid had a positive correlation with *Bacteroides* and *Escherichia*-*Shigella* and a negative correlation with f_*Lachnospiraceae*_Unclassified and *Lachnospiraceae*_NK4A136_group. *Bacteroides, Prevotellaceae*_Ga6A1_group, and *Alloprevotella* were negatively correlated with D-glucose and D-lactic acid and positively correlated with glycerol. While f_*Ruminococcaceae*_Unclassified had a positive correlation with D-glucose and D-lactic acid, it had a negative correlation with glycerol.

In addition, between the fecal metabolites and gut microbiota (Figure 8B), D-lactic acid was positively correlated with *Prevotella* and *Bacteroides*. Glycerol had a positive correlation with *Lactobacillus* and f_*Ruminococcaceae*_Unclassified and a negative correlation with *Bacteroides* and *Alloprevotella*. Inositol had a positive correlation with f_*Muribaculaceae*_Unclassified and f_*Lachnospiraceae*_Unclassified.

## Discussion

*Gynura segetum* is known to have various medicinal properties, especially for the treatment of cancer, diabetes, and hypertension. A study on the pharmacological effects and related chemical constituents of *Gynura segetum* found that 4, 5, 4′-trihydroxychalcone, 8, 8′-(ethene-1, 2-diyl)-dinaphtalene-1, 4, 5-triol, and rutin in the methanol extract of the plant had strong immunosuppressive effects (Yuandani and Husain, 2017). Another study on methanol extracts of *Gynura segetum* leaves showed that the extracts exhibited good antioxidant activity, which was related to the total phenolic and flavonoid content (Seow et al., 2014). Currently, PAs are the main toxic substances in the research on the chemical constituents of *Gynura segetum* causing hepatotoxicity. A study by Qi et al. (2009), identified and preliminarily characterized 20 compounds in the whole plant of *Gynura segetum* based on mass spectrometry and the possible biosynthetic pathways. Three PAs and one of the corresponding N-oxides, namely, seneciphylline, senecionine, and seneciphylline N-oxide, may be the material basis for the toxic effects of the plant (Qi et al., 2009). PAs are pro-toxins, and metabolic activation is a precondition for PA-induced toxicities (Cheeke, 1988). Most of the PA-induced cytotoxicity occurs in the liver, and an experiment based on human hepatic sinusoidal endothelial cells confirmed that PA-induced liver injury occurs mainly in hepatic sinusoidal endothelial cells (Yang et al., 2016). In addition, another study by Guo et al. (2021) on primary hepatocytes of rats found that PA-induced endoplasmic reticulum stress in hepatocytes may also be associated with its hepatotoxicity.

Drug-induced liver injury is a toxic damage or an allergic reaction caused by the drug itself or its metabolites. In recent years, its incidence has risen sharply and has become one of the most difficult major diseases to prevent and treat in the world. With the widespread use of Chinese herbal medicines worldwide, hepatotoxicity caused by Chinese herbal medicines has also become an important aspect of drug-induced liver injury. In addition to those herbal medicines containing PA, other herbal ingredients may also cause hepatotoxicity (Liu et al., 2019), such as psoralen and isopsoralen in *Psoralea corylifolia*; the main active ingredient rhein in *Polygonum multiflorum*; and evodiamine in Evodia fruit. At present, the clinical diagnosis and evaluation of drug-induced liver injury mainly rely on the Roussel Uclaf Causality Assessment Method (RUCAM). For example, Chow et al. (2019) showed the use of RUCAM to establish a causal relationship to the liver toxicity caused by Tusanqi (level 4). However, due to the complex composition of Chinese herbal medicines, the clinical manifestations and severity of hepatotoxicity caused by them vary widely, and the RUCAM scoring system may be helpful to identify them.

Even though studies reported that GSrE has a definite therapeutic effect on traumatic injury, it is undeniable that GSrE shows hepatotoxicity and there are many related reports. However, few studies reported the mechanism of hepatotoxicity induced by GSrE to the organism. In our present study, rats were given GSrE by gavage with different doses (7.5 and 15 g/kg) and exposure durations (10 and 21 days) to simulate the toxicological mechanism of the consumption of GSrE. Then, we combined urine and fecal metabolomics and 16S rDNA gene sequencing to investigate the effect of GSrE-induced hepatotoxicity on the whole body.

A study by Pannala et al. (2020) showed that abnormal amino acid metabolism is common in liver injury. From the metabolic pathway analysis, alanine, aspartate, and glutamate metabolisms; phenylalanine, tyrosine and tryptophan biosynthesis; and phenylalanine metabolism were the disturbed pathways in the urine and fecal metabolomics and were related to time and dose. In the urine metabolites, the levels of L-valine, l-alanine, L-proline, and L-lysine in the GSrE administration groups were always lower than those in the blank group, although the levels increased with the increase of time after administration. While L-valine, l-alanine, and L-lysine in fecal metabolomics decreased after administration, L-proline increased. Alanine and proline are the most frequently reported metabolites that are altered under toxicological impulses (Cuykx et al., 2018). The level of L-aspartic acid was increased after GSrE administration both in the urine and fecal metabolomics. L-aspartic acid is a non-essential amino acid, which shows a therapeutic effect on acute and chronic hepatitis and liver cirrhosis by regulating nitrogen metabolism, nucleic acid synthesis, and tricarboxylic acid cycle (Leng et al., 2014; Hou et al., 2017). Rao et al. (2021) showed that elevated L-aspartic acid concentration in the liver had beneficial effects on some fatty liver diseases. In fecal metabolomics, the level of phenylalanine increased after low-dose administration both on day 10 and day 21. Phenylalanine was identified as a potential biomarker of drug-induced hepatotoxicity with metabolomics analysis (Li et al., 2020a; Tu et al., 2021).

Disturbances in carbohydrate metabolism were found in the urine and fecal metabolomic results. Studies conducted by Nakajima et al. (1982) and Korourian et al. (1999) found that low carbohydrate intake would exacerbate liver damage or liver necrosis induced by alcohol or carbon tetrachloride. The pathway pentose and glucuronate interconversions were disturbed both in the urine and fecal samples, which could be seen in the toxicity model put forth by Zhou et al. (2021). Some polysaccharides containing DL-arabinose and D-rhamnose have been proved to have antioxidation and hepatoprotective effects (Xu et al., 2018; Kong et al., 2021). In the urine metabolomics, the content of DL-arabinose decreased after administration of high and low doses of GSrE both on days 10 and 21. The level of D-glucose in the urine metabolites in the low-dose groups increased with the advance of time, while the level showed a significant decrease in the high-dose groups. A study by Zanobbio et al. (2009) found that administered D-glucose exerts protective effects in different models of acute liver injury, which may due to the upregulation of anti-inflammatory factors. D-Allose is a rare monosaccharide that has a protective effect on liver ischemia-reperfusion injury (Hossain et al., 2003), but in our results, its level was always higher than that of the blank group and even increased significantly after high-dose administration.

D-lactic acid, the significantly changed differential metabolite, decreased in both urine and fecal metabolomics results. A study by Geng et al. (2020) showed that disturbed D-lactic acid metabolism means that both aerobic and anaerobic processes of energy metabolism were impaired. In addition, SCFAs also play a role in the models of hepatotoxicity. For example, butyrate has a detoxification effect on a genipin-induced liver injury by promoting colonic integrity and promoting Nrf2 activation (Luo et al., 2021). A study by Mun et al. (2021) showed that an SCFA mixture treatment improved metabolic activation of liver organoids and contributed to the accurate evaluation of the CYP3A4-dependent drug toxicity. In our metabolomics results, the level of pentanoic acid in urine was decreased after GSrE-induced hepatotoxicity, and the level on day 21 was lower than that on day 10. The level of butanoic acid of GSrE administration in feces was lower than the blank group, which indicated reduced SCFAs in GSrE-induced hepatotoxicity rats.

Abnormal lipid metabolism is common in hepatotoxicity and even acts as a marker of liver injury (Huang et al., 2020; Cheng et al., 2022). Glycerol is a small molecule that is an important intermediate between carbohydrate and lipid metabolism. In the liver, glycerol acts as a gluconeogenic precursor and esterifies free fatty acids to triglycerides (Lebeck, 2014). In our results, the level of glycerol was increased both in the urine and feces after GSrE-induced hepatotoxicity. It has been reported that intraperitoneal glycerol could induce hepatotoxicity characterized by a dramatic increase in plasma ALT (Zager, 2015). The level of inositol was increased in the GSrE-induced hepatotoxicity in our urine and fecal metabolomics results. Inositol is a ubiquitous cyclic alcohol with important regulatory roles. Inositol supplementation reduces hepatic triglyceride and cholesterol accumulation in animal models of fatty liver (Pani et al., 2020).

GSrE-induced hepatotoxicity also significantly alters the composition of the gut microbiota. Similar to our results, a low abundance of f_*Muribaculaceae*_Unclassified was often seen in the models of nonalcoholic fatty liver disease, obesity, liver steatosis, and sepsis-related liver injury (Xia et al., 2021; Yuan et al., 2021; Liang et al., 2022). Chen et al. (2018), reports that *Lactobacillus* has hepatoprotective effects. *Lactobacillus* improves the progression of nonalcoholic steatosis by lowering cholesterol (Lee et al., 2021). Our results also showed a decreased abundance of *Lactobacillus* in GSrE-induced hepatotoxicity rats and showed a dose-dependence on day 10 and a time-dependence in low-dose groups. Our results showed a decreased abundance of *Bacteroides* in the hepatotoxicity rats, and there has been evidence that alcohol-induced liver injury mice had an extremely low proportion of *Bacteroides* Ferrere et al. (2017). Bacteroides can break down complex polysaccharides, participate in immune metabolism, and play a role in preventing obesity; however, it is also a pathogen that can cause many infectious diseases (Wexler, 2007). Hu et al. (2019) reported that the *Lachnospiraceae*_NK4A136_group is a butyrate-producing bacterium that enhances epithelial barrier integrity and inhibits inflammation. A study by Zha et al. (2022) found that the *Lachnospiraceae*_NK4A136_group had a protective effect on D-GalN-induced rat liver injury, though in our results, the abundance of the bacterium raised after GSrE-induced hepatotoxicity. f_*Ruminococcaceae*_Unclassified is a dominant bacterium in the nonalcoholic fatty liver disease and positively correlated with inflammation and hepatic steatosis-related factors, as was also observed in studies by Guo et al. (2018) and Hu et al. (2021b). The *Prevotellaceae*_Ga6A1_group is often seen in those on high-sugar diets and those with diabetes, and hepatic glucose and lipid metabolic disorders may cause liver diseases according to Yue et al. (2019) and Yang et al. (2021). *Escherichia*-*Shigella* is positively correlated with hepatic inflammatory factors, which further stimulate liver injury and inflammation (Liang et al., 2022). f_*Ruminococcaceae*_Unclassified, *Prevotellaceae*_Ga6A1_group, and *Escherichia*-*Shigella* were all increased after GSrE-induced hepatotoxicity in our results.

With the correlation analysis between gut microbiota and the metabolites in urine and feces, the tight crosstalk between the gut microbiota and host metabolism in GSrE-induced hepatotoxicity were highlighted. The gut microbiota participates in various metabolic processes of the body involving energy, lipid, and amino acid metabolisms. Interacting with metabolites is one of the major ways that gut microbiota affect the host, from which microbial metabolites have many biological functions, such as regulating immune homeostasis, host energy metabolism, and participating in various disease processes (Usami et al., 2015; Lavelle and Sokol, 2020). The crosstalk between gut microbiota and metabolites in this study explains part of the mechanism of metabolic disturbances by GSrE-induced hepatotoxicity.

## Conclusion

In summary, urine and fecal metabolomic analyses revealed that oral administration of different doses of GSrE for 10 and 21 days induced the hepatotoxicity-disrupted peripheral metabolism. Differential metabolites in urine and feces involved amino acids, carbohydrates, lipids, organic acids, and SCFAs, among others. Among them, L-valine, L-proline, DL-arabinose, pentanoic acid, D-allose, and D-glucose in urine and D-lactic acid and glycerol in feces were time- and dose-related differential metabolites. Furthermore, the 16S rDNA sequencing analysis revealed that GSrE-induced hepatotoxicity associated with the time and dose of administration significantly altered the composition of gut microbiota. Finally, the results of correlation analysis revealed the close crosstalk between gut microbiota and differential metabolites after the administration of GSrE to induce hepatotoxicity, and gut microbiota may have participated in amino acid, energy, and lipid metabolisms.

## Data availability statement

The datasets presented in this study can be found in online repositories. The names of the repository/repositories and accession number(s) can be found below: NCBI—PRJNA851539.

## Ethics statement

The animal study was reviewed and approved by Animal Experiment Center, Shanghai University of Traditional Chinese Medicine.

## Author contributions

XG analyzed the data and wrote the manuscript. XG, SL, and ML carried out the research. LC and YJ helped to develop the experimental idea and design. QW and YL conducted the animal experiments. XG and SC were responsible for metabolomics studies and the analysis of the data. TZ and MZ revised the manuscript. MZ and XG designed and funded the experiment and approved the publication of the final version of the manuscript. All authors contributed to the article and approved the submitted version.

## Funding

This project was supported by the Baoshan District Hospital of Integrated Traditional Chinese and Western Medicine of Shanghai combined with the National Natural Education fund (No. GZRPYJJ-201605) and the Innovation Project for Undergraduates of Shanghai University of Traditional Chinese Medicine (202210268241).

## Conflict of interest

The authors declare that the research was conducted in the absence of any commercial or financial relationships that could be construed as a potential conflict of interest.

## Publisher's note

All claims expressed in this article are solely those of the authors and do not necessarily represent those of their affiliated organizations, or those of the publisher, the editors and the reviewers. Any product that may be evaluated in this article, or claim that may be made by its manufacturer, is not guaranteed or endorsed by the publisher.

## Abbreviations

DHPA: dehydropyrrolizidine alkaloid
GC-MS: gas chromatography-mass spectrometry
GSrE: *Gynura segetum* (Lour.) Merr. root extract
OPLS-DA: orthogonal partial least squares-discriminant analysis
OTU: operational taxonomic unit
PAs: pyrrolizidine alkaloids
PCA: principal component analysis
SCFAs: short chain fatty acids
VIP: variable importance in the project.
