# Supplementary material for: Investigation of Gynura segetum root extract (GSrE) induced hepatotoxicity based on metabolomic signatures and microbial community profiling in rats
Source: Front Microbiol. 2022 Aug 9;13:947757. doi: 10.3389/fmicb.2022.947757 (PMC9396145; doi:10.3389/fmicb.2022.947757)
Supplement: Supplementary file 1 [file Data_Sheet_1.docx]

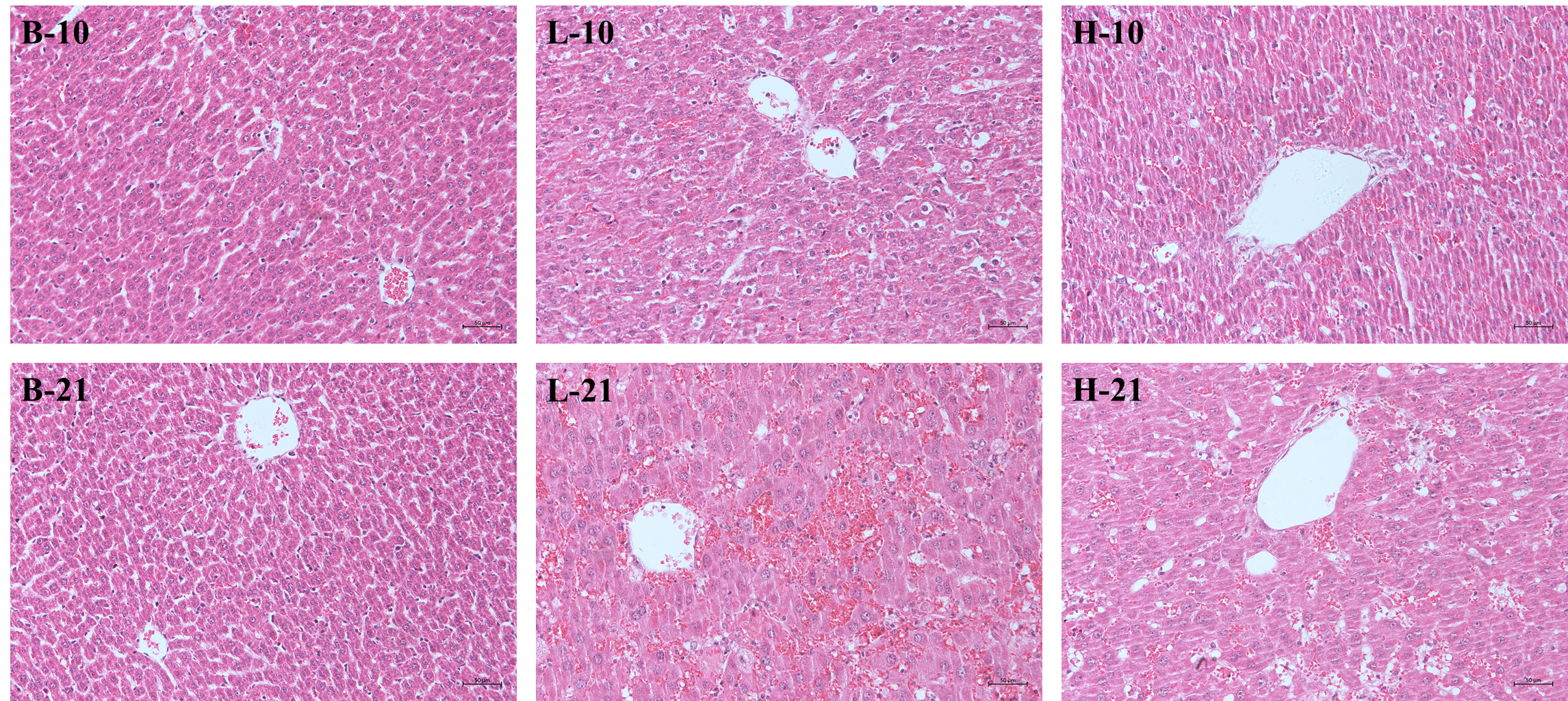


**Figure S1** Hematoxylin and Eosin (H&E) staining (200×) of liver tissue in SD rats treated with different dose of GSrE at 10th and 21st day.

**Table S1** The level of serum ALT and AST in each group.

| Groups | ALT (U/L) | AST (U/L) |
| --- | --- | --- |
| B-10 | 27.50 ± 2.93 | 100.50 ± 6.89 |
| B-21 | 23.88 ± 3.68 | 97.50 ± 7.87 |
| L-10 | 207.33 ± 63.53^**^ | 374.67 ± 191.97^*^ |
| L-21 | 217.67 ± 100.33^**^ | 378.20 ± 185.17^**^ |
| H-10 | 415.20 ± 224.16^**^ | 321.50 ± 115.11^*^ |
| H-21 | 243.67 ± 59.41^**^ | 459.00 ± 115.00^**^ |

Notes: The serum ALT and AST analysis was carried out with ELISA kits. * P < 0.05, ** P < 0.01, compared with the corresponding blank group. Values were represented as mean ± SD (n=4-8).

**Table S2** The differential metabolites of urine samples between different dose groups at the same day.

| No | Metabolites | RT  (min) | Mass  (*m/z*) | group | VIP value ^a^ | *P* value | FC ^b^ | Trend |
| --- | --- | --- | --- | --- | --- | --- | --- | --- |
| 1 | Thiourea | 5.79 | 171.00 | B-21 vs L-21 | 1.60 | 0.02 | 1.56 | ↑ |
|  |  |  |  | B-21 vs H-21 | 2.51 | 0.02 | 2.46 | ↑ |
| 2 | D-Lactic acid | 7.01 | 59.09 | B-10 vs H-10 | 1.65 | 0.04 | 1.65 | ↑ |
|  |  |  |  | B-21 vs L-21 | 1.88 | 0.01 | 1.71 | ↑ |
| 3 | Hexanoic acid | 7.06 | 75.06 | B-10 vs H-10 | 2.74 | 0.03 | 1.81 | ↑ |
| 4 | L-Valine | 7.40 | 55.09 | B-10 vs L-10 | 2.48 | 0.00 | 2.38 | ↑ |
|  |  |  |  | B-10 vs H-10 | 1.72 | 0.01 | 1.84 | ↑ |
|  |  |  |  | B-21 vs L-21 | 1.62 | 0.01 | 1.59 | ↑ |
| 5 | D-Alanine | 7.43 | 72.09 | B-10 vs L-10 | 5.33 | 0.00 | 3.83 | ↑ |
|  |  |  |  | B-10 vs H-10 | 3.15 | 0.02 | 1.86 | ↑ |
| 6 | Acetic acid | 8.08 | 86.07 | B-10 vs L-10 | 4.74 | 0.01 | 4.34 | ↑ |
| 7 | Phosphoric acid | 8.63 | 68.08 | B-21 vs H-21 | 1.04 | 0.01 | 3.81 | ↑ |
| 8 | Pentanoic acid | 8.64 | 70.09 | B-10 vs L-10 | 3.83 | 0.01 | 3.91 | ↑ |
|  |  |  |  | B-21 vs L-21 | 1.54 | 0.00 | 2.77 | ↑ |
|  |  |  |  | B-21 vs H-21 | 1.76 | 0.01 | 3.17 | ↑ |
| 9 | α-D-Galactopyranose | 10.01 | 103.00 | B-10 vs H-10 | 2.21 | 0.00 | 0.63 | ↓ |
| 10 | Glycerol | 10.07 | 72.05 | B-10 vs L-10 | 1.19 | 0.01 | 0.56 | ↓ |
|  |  |  |  | B-21 vs H-21 | 1.69 | 0.02 | 0.57 | ↓ |
| 11 | D-Threonine | 10.08 | 298.08 | B-21 vs L-21 | 2.73 | 0.01 | 3.08 | ↑ |
| 12 | L-Isoleucine | 10.11 | 219.00 | B-10 vs H-10 | 1.11 | 0.03 | 0.45 | ↓ |
| 13 | Propanoic acid | 10.77 | 113.00 | B-10 vs H-10 | 1.06 | 0.04 | 2.07 | ↑ |
| 14 | Pyrimidine | 10.86 | 255.00 | B-10 vs H-10 | 1.63 | 0.03 | 2.26 | ↑ |
| 15 | Pentanedioic acid | 11.57 | 101.00 | B-10 vs H-10 | 1.01 | 0.02 | 2.92 | ↑ |
| 16 | Butanoic acid | 12.40 | 243.03 | B-10 vs H-10 | 1.01 | 0.04 | 4.04 | ↑ |
| 17 | L-Proline | 13.14 | 128.00 | B-10 vs L-10 | 4.72 | 0.03 | 1.12 | ↑ |
|  |  |  |  | B-10 vs H-10 | 1.27 | 0.00 | 2.95 | ↑ |
|  |  |  |  | B-21 vs L-21 | 4.79 | 0.04 | 0.54 | ↓ |
| 18 | L-Aspartic acid | 13.17 | 100.00 | B-10 vs L-10 | 1.68 | 0.01 | 0.58 | ↓ |
|  |  |  |  | B-21 vs L-21 | 1.70 | 0.02 | 0.42 | ↓ |
| 19 | Gluconic acid | 13.24 | 147.00 | B-10 vs L-10 | 3.16 | 0.03 | 0.39 | ↓ |
| 20 | Benzoic acid | 13.62 | 73.10 | B-21 vs L-21 | 2.54 | 0.05 | 1.56 | ↑ |
| 21 | Benzeneacetic acid | 14.44 | 192.00 | B-10 vs H-10 | 1.59 | 0.00 | 3.81 | ↑ |
| 22 | D-Xylose | 14.86 | 308.10 | B-21 vs L-21 | 1.90 | 0.03 | 4.15 | ↑ |
| 23 | DL-Arabinose | 14.87 | 73.07 | B-10 vs L-10 | 7.37 | 0.03 | 1.93 | ↑ |
|  |  |  |  | B-10 vs H-10 | 7.08 | 0.03 | 2.53 | ↑ |
|  |  |  |  | B-21 vs L-21 | 8.96 | 0.00 | 3.26 | ↑ |
| 24 | Inosose | 14.92 | 189.00 | B-10 vs L-10 | 2.64 | 0.02 | 3.29 | ↑ |
|  |  |  |  | B-21 vs L-21 | 1.91 | 0.01 | 2.14 | ↑ |
| 25 | L-Fucose | 15.47 | 147.00 | B-10 vs H-10 | 1.07 | 0.00 | 2.90 | ↑ |
| 26 | D-Rhamnose | 15.58 | 117.00 | B-10 vs L-10 | 3.97 | 0.03 | 2.25 | ↑ |
| 27 | D-Allose | 17.34 | 191.00 | B-10 vs H-10 | 1.98 | 0.04 | 0.25 | ↓ |
|  |  |  |  | B-21 vs L-21 | 1.36 | 0.05 | 0.22 | ↓ |
|  |  |  |  | B-21 vs H-21 | 1.51 | 0.02 | 0.26 | ↓ |
| 28 | D-Glucose | 17.44 | 157.00 | B-10 vs H-10 | 1.82 | 0.03 | 0.48 | ↓ |
|  |  |  |  | B-21 vs L-21 | 2.80 | 0.00 | 0.29 | ↓ |
|  |  |  |  | B-21 vs H-21 | 2.31 | 0.04 | 0.29 | ↓ |
| 29 | L-Tyrosine | 17.46 | 73.08 | B-21 vs L-21 | 7.51 | 0.04 | 0.44 | ↓ |
|  |  |  |  | B-21 vs H-21 | 7.04 | 0.05 | 0.54 | ↓ |
| 30 | L-Lysine | 17.51 | 127.99 | B-10 vs L-10 | 1.91 | 0.03 | 3.29 | ↑ |
|  |  |  |  | B-10 vs H-10 | 1.19 | 0.02 | 2.18 | ↑ |
|  |  |  |  | B-21 vs L-21 | 1.01 | 0.00 | 4.35 | ↑ |
|  |  |  |  | B-21 vs H-21 | 1.07 | 0.01 | 4.06 | ↑ |
| 31 | D-Talose | 17.54 | 233.00 | B-10 vs H-10 | 1.05 | 0.01 | 0.36 | ↓ |
| 32 | Inositol | 18.05 | 319.17 | B-10 vs H-10 | 2.15 | 0.02 | 0.00 | ↓ |
|  |  |  |  | B-21 vs L-21 | 1.58 | 0.04 | 0.30 | ↓ |
|  |  |  |  | B-21 vs H-21 | 2.07 | 0.03 | 0.27 | ↓ |
| 33 | N-Acetyl-D-glucosamine | 19.10 | 69.05 | B-10 vs H-10 | 1.18 | 0.01 | 0.24 | ↓ |
|  |  |  |  | B-21 vs L-21 | 1.92 | 0.04 | 0.20 | ↓ |
|  |  |  |  | B-21 vs H-21 | 1.96 | 0.04 | 0.25 | ↓ |
| 34 | Uridine | 20.52 | 406.10 | B-21 vs H-21 | 1.37 | 0.05 | 0.03 | ↓ |
| 35 | Sebacic acid | 21.80 | 331.05 | B-21 vs L-21 | 1.02 | 0.03 | 0.12 | ↓ |
| 36 | 5-Methyluridine | 21.90 | 245.99 | B-21 vs L-21 | 1.12 | 0.02 | 0.12 | ↓ |
| 37 | Maltose | 24.07 | 450.20 | B-10 vs L-10 | 1.38 | 0.02 | 9.08 | ↑ |
|  |  |  |  | B-21 vs L-21 | 1.35 | 0.04 | 0.18 | ↓ |

Note: ^a^ VIP value was obtained from OPLS-DA. ^b^ Fold change (FC) was calculated as the ratio of the average relative level between two groups (FC value=B-10/L-10 or B-10/H-10 or B-21/L-21 or B-21/H-21). ^c^ Trend: ↑ Metabolites increased in B-10 or B-21 group; ↓ Metabolites decreased in B-10 or B-21 group.

**Table S3** The differential metabolites of urine samples at different day in the same dose groups.

| No | Metabolites | RT  (min) | Mass  (*m/z*) | group | VIP value ^a^ | *P* value | FC ^b^ | Trend |
| --- | --- | --- | --- | --- | --- | --- | --- | --- |
| 1 | Hexanoic acid | 7.06 | 75.06 | L-0 vs L-10 | 1.35 | 0.00 | 5.50 | ↑ |
|  |  |  |  | L-0 vs L-21 | 1.32 | 0.00 | 4.46 | ↑ |
|  |  |  |  | H-0 vs H-21 | 1.00 | 0.00 | 0.01 | ↓ |
| 2 | L-Valine | 7.40 | 55.09 | L-0 vs L-10 | 1.46 | 0.00 | 0.03 | ↓ |
|  |  |  |  | L-0 vs L-21 | 2.71 | 0.00 | 0.01 | ↓ |
|  |  |  |  | H-0 vs H-10 | 2.30 | 0.00 | 0.02 | ↓ |
|  |  |  |  | H-0 vs H-21 | 2.48 | 0.00 | 0.01 | ↓ |
| 3 | L-Alanine | 7.43 | 72.09 | L-0 vs L-10 | 4.84 | 0.00 | 0.03 | ↓ |
|  |  |  |  | L-0 vs L-21 | 4.68 | 0.00 | 0.03 | ↓ |
|  |  |  |  | H-0 vs H-10 | 3.43 | 0.00 | 0.03 | ↓ |
|  |  |  |  | H-0 vs H-21 | 3.27 | 0.02 | 0.03 | ↓ |
| 4 | Ethanedioic acid | 8.06 | 147.06 | L-0 vs L-10 | 1.39 | 0.03 | 1.86 | ↑ |
|  |  |  |  | L-0 vs L-21 | 2.30 | 0.00 | 5.57 | ↑ |
|  |  |  |  | H-0 vs H-10 | 2.01 | 0.00 | 0.17 | ↓ |
| 5 | Acetic acid | 8.08 | 86.07 | L-0 vs L-10 | 1.25 | 0.00 | 0.30 | ↓ |
|  |  |  |  | L-0 vs L-21 | 1.21 | 0.00 | 0.31 | ↓ |
|  |  |  |  | H-0 vs H-10 | 1.03 | 0.00 | 0.39 | ↓ |
|  |  |  |  | H-0 vs H-21 | 1.76 | 0.00 | 0.20 | ↓ |
| 6 | Pentanoic acid | 8.64 | 70.09 | L-0 vsL-10 | 1.83 | 0.00 | 0.14 | ↓ |
|  |  |  |  | L-0 vs L-21 | 1.51 | 0.00 | 0.20 | ↓ |
|  |  |  |  | H-0 vs H-10 | 1.60 | 0.00 | 0.17 | ↓ |
|  |  |  |  | H-0 vs H-21 | 1.84 | 0.00 | 0.15 | ↓ |
| 7 | L-Norvaline | 9.18 | 144.00 | L-0 vsL-10 | 2.73 | 0.00 | 0.01 | ↓ |
|  |  |  |  | L-0 vs L-21 | 1.54 | 0.03 | 0.01 | ↓ |
| 8 | L-Threose | 9.98 | 129.00 | H-0 vs H-10 | 1.09 | 0.00 | 0.06 | ↓ |
|  |  |  |  | H-0 vs H-21 | 1.09 | 0.00 | 0.06 | ↓ |
| 9 | Phosphate | 10.04 | 299.04 | H-0 vs H-10 | 7.33 | 0.00 | 3.83 | ↑ |
|  |  |  |  | H-0 vs H-21 | 6.26 | 0.02 | 2.63 | ↑ |
| 10 | L-Isoleucine | 10.11 | 219.00 | L-0 vsL-10 | 1.80 | 0.00 | 0.05 |  |
|  |  |  |  | L-0 vs L-21 | 1.10 | 0.01 | 0.09 | ↓ |
| 11 | Butanedioic acid | 10.41 | 174.09 | L-0 vsL-10 | 2.16 | 0.00 | 0.33 | ↓ |
|  |  |  |  | L-0 vs L-21 | 2.75 | 0.00 | 0.24 | ↓ |
|  |  |  |  | H-0 vs H-10 | 1.08 | 0.00 | 7.07 | ↑ |
|  |  |  |  | H-0 vs H-21 | 1.11 | 0.01 | 2.89 | ↑ |
| 12 | Propanoic acid | 10.77 | 113.00 | L-0 vsL-10 | 1.57 | 0.00 | 0.18 | ↓ |
|  |  |  |  | L-0 vs L-21 | 1.48 | 0.00 | 0.19 | ↓ |
|  |  |  |  | H-0 vs H-10 | 2.06 | 0.01 | 0.23 | ↓ |
| 13 | Pyrimidine | 10.86 | 255.00 | L-0 vsL-10 | 1.15 | 0.00 | 0.16 | ↓ |
|  |  |  |  | L-0 vs L-21 | 1.08 | 0.00 | 0.16 | ↓ |
| 14 | Pentanedioic acid | 11.57 | 101.00 | L-0 vsL-10 | 1.06 | 0.00 | 0.04 | ↓ |
|  |  |  |  | L-0 vs L-21 | 1.11 | 0.00 | 0.03 | ↓ |
|  |  |  |  | H-0 vs H-10 | 2.72 | 0.00 | 0.10 | ↓ |
|  |  |  |  | H-0 vs H-21 | 3.17 | 0.00 | 0.08 | ↓ |
| 15 | Butanoic acid | 12.40 | 243.03 | L-0 vs L-21 | 1.92 | 0.00 | <0.00 | ↓ |
|  |  |  |  | H-0 vs H-10 | 2.29 | 0.01 | 3.25 | ↑ |
|  |  |  |  | H-0 vs H-21 | 2.25 | 0.04 | 3.33 | ↑ |
| 16 | L-Methionine | 12.51 | 100.00 | L-0 vs L-21 | 1.06 | 0.00 | 3.98 | ↑ |
| 17 | Aminomalonic acid | 12.52 | 254.89 | H-0 vs H-10 | 1.24 | 0.00 | 5.37 | ↑ |
|  |  |  |  | H-0 vs H-21 | 1.22 | 0.00 | 5.29 | ↑ |
| 18 | L-Threitol | 12.88 | 217.10 | L-0 vs L-10 | 1.02 | 0.00 | 1.39 | ↑ |
|  |  |  |  | L-0 vs L-21 | 1.01 | 0.00 | 2.54 | ↑ |
|  |  |  |  | H-0 vs H-10 | 1.00 | 0.00 | 6.85 | ↑ |
|  |  |  |  | H-0 vs H-21 | 1.08 | 0.00 | 2.79 | ↑ |
| 19 | L-Proline | 13.14 | 128.00 | L-0 vsL-10 | 5.11 | 0.00 | 0.08 | ↓ |
|  |  |  |  | L-0 vs L-21 | 4.64 | 0.00 | 0.09 | ↓ |
|  |  |  |  | H-0 vs H-10 | 1.44 | 0.00 | 0.01 | ↓ |
|  |  |  |  | H-0 vs H-21 | 1.96 | 0.01 | 0.00 | ↓ |
| 20 | L-Aspartic acid | 13.17 | 100.00 | L-0 vsL-10 | 4.67 | 0.00 | 0.11 | ↓ |
|  |  |  |  | L-0 vs L-21 | 4.64 | 0.00 | 0.10 | ↓ |
| 21 | Gluconic acid | 13.24 | 147.00 | L-0 vsL-10 | 1.26 | 0.00 | 3.15 | ↑ |
|  |  |  |  | L-0 vs L-21 | 1.24 | 0.00 | 4.66 | ↑ |
|  |  |  |  | H-0 vs H-10 | 1.04 | 0.00 | 8.87 | ↑ |
|  |  |  |  | H-0 vs H-21 | 1.08 | 0.00 | 9.50 | ↑ |
| 22 | L-Threonic acid | 13.61 | 171.99 | L-0 vsL-10 | 2.63 | 0.00 | 2.74 | ↑ |
|  |  |  |  | L-0 vs L-21 | 2.49 | 0.00 | 2.51 | ↑ |
|  |  |  |  | H-0 vs H-10 | 1.18 | 0.01 | 6.65 | ↑ |
|  |  |  |  | H-0 vs H-21 | 1.15 | 0.03 | 7.09 | ↑ |
| 23 | Benzoic acid | 13.62 | 73.10 | H-0 vs H-10 | 1.17 | 0.00 | 5.40 | ↑ |
|  |  |  |  | H-0 vs H-21 | 1.18 | 0.00 | 6.78 | ↑ |
| 24 | 2-Deoxy-D-ribose | 13.87 | 116.00 | H-0 vs H-10 | 1.00 | 0.00 | 3.19 | ↑ |
|  |  |  |  | H-0 vs H-21 | 1.00 | 0.00 | 3.15 | ↑ |
| 25 | Glutamic acid | 14.20 | 146.05 | H-0 vs H-10 | 1.23 | 0.00 | 1.10 | ↑ |
|  |  |  |  | H-0 vs H-21 | 1.28 | 0.00 | 4.54 | ↑ |
| 26 | Benzeneacetic acid | 14.44 | 192.00 | H-0 vs H-10 | 1.39 | 0.00 | 0.09 | ↓ |
|  |  |  |  | H-0 vs H-21 | 1.05 | 0.01 | 0.14 | ↓ |
| 27 | D-Xylose | 14.86 | 308.10 | L-0 vs L-10 | 2.08 | 0.00 | 0.01 | ↓ |
|  |  |  |  | L-0 vs L-21 | 2.16 | 0.00 | 0.01 | ↓ |
| 28 | DL-Arabinose | 14.87 | 73.07 | L-0 vs L-10 | 1.36 | 0.01 | 0.14 | ↓ |
|  |  |  |  | L-0 vs L-21 | 1.35 | 0.00 | 0.16 | ↓ |
|  |  |  |  | H-0 vs H-10 | 2.05 | 0.00 | 0.20 | ↓ |
|  |  |  |  | H-0 vs H-21 | 2.19 | 0.00 | 0.19 | ↓ |
| 29 | Xylitol | 15.35 | 217.10 | L-0 vs L-10 | 1.12 | 0.00 | 1.82 | ↑ |
|  |  |  |  | L-0 vs L-21 | 1.09 | 0.00 | 9.88 | ↑ |
|  |  |  |  | H-0 vs H-10 | 1.08 | 0.02 | 5.10 | ↑ |
|  |  |  |  | H-0 vs H-21 | 1.02 | 0.05 | 4.71 | ↑ |
| 30 | d-Mannose | 15.47 | 147.00 | L-0 vs L-10 | 1.93 | 0.00 | 6.86 | ↑ |
|  |  |  |  | L-0 vs L-21 | 1.96 | 0.00 | 1.70 | ↑ |
|  |  |  |  | H-0 vs H-10 | 2.20 | 0.00 | 2.65 | ↑ |
|  |  |  |  | H-0 vs H-21 | 2.14 | 0.00 | 5.17 | ↑ |
| 31 | D-Rhamnose | 15.58 | 117.00 | L-0 vs L-10 | 2.22 | 0.00 | 0.05 | ↓ |
|  |  |  |  | L-0 vs L-21 | 1.11 | 0.00 | 0.13 | ↓ |
| 32 | Propanoate | 15.99 | 218.00 | L-0 vs L-10 | 2.67 | 0.00 | 0.05 | ↓ |
|  |  |  |  | L-0 vs L-21 | 1.33 | 0.00 | 0.15 | ↓ |
| 33 | L-Ornithine | 16.42 | 149.00 | L-0 vs L-10 | 1.03 | 0.00 | 4.56 | ↑ |
|  |  |  |  | L-0 vs L-21 | 1.12 | 0.00 | 1.45 | ↑ |
| 34 | 1,2,3-Propanetricarboxylic acid | 16.53 | 375.10 | L-0 vs L-10 | 1.72 | 0.00 | 5.00 | ↑ |
|  |  |  |  | L-0 vs L-21 | 1.64 | 0.00 | 2.51 | ↑ |
|  |  |  |  | H-0 vs H-10 | 3.44 | 0.00 | 9.37 | ↑ |
|  |  |  |  | H-0 vs H-21 | 3.45 | 0.00 | 3.94 | ↑ |
| 35 | 2-Furanacetaldehyde | 16.98 | 91.03 | H-0 vs H-10 | 1.36 | 0.00 | 4.87 | ↑ |
|  |  |  |  | H-0 vs H-21 | 1.36 | 0.00 | 8.83 | ↑ |
| 36 | D-Fructose | 17.24 | 178.99 | H-0 vs H-10 | 1.09 | 0.02 | 0.05 | ↓ |
| 37 | D-Allose | 17.34 | 191.00 | L-0 vs L-10 | 1.17 | 0.00 | 0.08 | ↓ |
|  |  |  |  | L-0 vs L-21 | 1.35 | 0.00 | 0.06 | ↓ |
|  |  |  |  | H-0 vs H-10 | 2.04 | 0.00 | 0.10 | ↓ |
|  |  |  |  | H-0 vs H-21 | 1.46 | 0.00 | 0.17 | ↓ |
| 38 | D-Glucose | 17.44 | 157.00 | L-0 vsL-10 | 1.19 | 0.00 | 0.54 | ↓ |
|  |  |  |  | L-0 vs L-21 | 1.31 | 0.00 | 0.47 | ↓ |
|  |  |  |  | H-0 vs H-10 | 1.40 | 0.01 | 6.12 | ↑ |
|  |  |  |  | H-0 vs H-21 | 1.56 | 0.02 | 6.35 | ↑ |
| 39 | L-Lysine | 17.51 | 127.99 | L-0 vsL-10 | 1.16 | 0.00 | 0.32 | ↓ |
|  |  |  |  | L-0 vs L-21 | 1.88 | 0.00 | 0.19 | ↓ |
| 40 | Lactulose | 17.93 | 333.10 | L-0 vsL-10 | 1.48 | 0.00 | 6.93 | ↑ |
|  |  |  |  | L-0 vs L-21 | 1.32 | 0.00 | 4.25 | ↑ |
| 41 | beta-D-Glucopyranosiduronic acid | 18.50 | 353.04 | L-0 vs L-21 | 1.10 | 0.00 | 0.02 | ↓ |
| 42 | N-Acetyl glucosamine methoxime | 18.96 | 259.09 | L-0 vsL-10 | 1.28 | 0.00 | 5.76 | ↑ |
|  |  |  |  | L-0 vs L-21 | 1.24 | 0.00 | 8.63 | ↑ |
|  |  |  |  | H-0 vs H-10 | 1.39 | 0.00 | 3.10 | ↑ |
|  |  |  |  | H-0 vs H-21 | 1.39 | 0.01 | 7.72 | ↑ |
| 43 | N-Acetyl-D-glucosamine | 19.10 | 69.05 | L-0 vs L-10 | 1.51 | 0.00 | 6.66 | ↑ |
| 44 | Uric acid | 19.26 | 73.07 | L-0 vs L-10 | 2.58 | 0.00 | 0.33 | ↓ |
|  |  |  |  | L-0 vs L-21 | 2.96 | 0.00 | 0.28 | ↓ |
|  |  |  |  | H-0 vs H-10 | 4.62 | 0.00 | 0.12 | ↓ |
|  |  |  |  | H-0 vs H-21 | 3.67 | 0.00 | 0.18 | ↓ |
| 45 | Octadecanoic acid | 21.18 | 217.10 | L-0 vs L-10 | 2.49 | 0.00 | 5.74 | ↑ |
|  |  |  |  | L-0 vs L-21 | 2.44 | 0.00 | 8.20 | ↑ |
|  |  |  |  | H-0 vs H-10 | 2.66 | 0.00 | 7.32 | ↑ |
|  |  |  |  | H-0 vs H-21 | 2.59 | 0.02 | 6.39 | ↑ |
| 46 | D-Turanose | 23.68 | 204.10 | H-0 vs H-10 | 2.74 | 0.00 | 9.89 | ↑ |
|  |  |  |  | H-0 vs H-21 | 2.67 | 0.00 | 6.93 | ↑ |
| 47 | Maltose | 24.07 | 450.20 | L-0 vs L-10 | 1.52 | 0.00 | 5.56 | ↑ |
|  |  |  |  | L-0 vs L-21 | 1.42 | 0.00 | 1.59 | ↑ |
|  |  |  |  | H-0 vs H-10 | 1.90 | 0.00 | 9.81 | ↑ |
|  |  |  |  | H-0 vs H-21 | 1.84 | 0.01 | 2.11 | ↑ |
| 48 | D-Lactose | 24.23 | 205.10 | H-0 vs H-10 | 2.70 | 0.00 | 9.69 | ↑ |
|  |  |  |  | H-0 vs H-21 | 2.71 | 0.00 | 7.64 | ↑ |

Note: ^a^ VIP value was obtained from OPLS-DA. ^b^ Fold change (FC) was calculated as the ratio of the average relative level between two groups (FC value=L-0/L-10 or L-0/L-21 or H-0/H-10 or H-0/H-21). ^c^ Trend: ↑ Metabolites increased in L-0 or H-0 group; ↓ Metabolites decreased in L-0 or H-0 group.

**Table S4** The differential metabolites of fecal samples between different dose groups at the same day.

| No | Metabolites | RT  (min) | Mass  (*m/z*) | group | VIP value ^a^ | *P* value | FC ^b^ | Trend |
| --- | --- | --- | --- | --- | --- | --- | --- | --- |
| 1 | Thiourea | 5.78 | 171.00 | B-21 vs L-21 | 1.32 | 0.03 | 1.64 | ↑ |
|  |  |  |  | B-21 vs H-21 | 2.01 | 0.02 | 2.57 | ↑ |
| 2 | Pentanoic acid | 5.90 | 70.09 | B-21 vs L-21 | 1.22 | 0.00 | 2.89 | ↑ |
|  |  |  |  | B-21 vs H-21 | 1.40 | 0.01 | 3.27 | ↑ |
| 3 | D-Lactic acid | 7.00 | 59.09 | B-10 vs H-10 | 1.30 | 0.03 | 1.68 | ↑ |
|  |  |  |  | B-21 vs L-21 | 1.56 | 0.00 | 1.77 | ↑ |
| 4 | Hexanoic acid | 7.06 | 75.06 | B-10 vs H-10 | 2.12 | 0.03 | 1.85 | ↑ |
| 5 | L-Valine | 7.33 | 55.09 | B-10 vs L-10 | 1.84 | 0.00 | 2.36 | ↑ |
|  |  |  |  | B-10 vs H-10 | 1.37 | 0.01 | 1.89 | ↑ |
|  |  |  |  | B-21 vs L-21 | 1.39 | 0.00 | 1.66 | ↑ |
|  |  |  |  | B-21 vs H-21 | 1.08 | 0.01 | 3.11 | ↑ |
| 6 | l-Alanine | 7.59 | 72.09 | B-10 vs H-10 | 4.81 | 0.03 | 2.26 | ↑ |
| 7 | Butanoic acid | 8.44 | 243.03 | B-10 vs L-10 | 3.65 | 0.01 | 4.29 | ↑ |
| 8 | Thymine | 8.61 | 70.09 | B-10 vs L-10 | 2.94 | 0.01 | 3.81 | ↑ |
| 9 | Phosphoric acid | 8.62 | 171.98 | B-21 vs H-21 | 3.40 | 0.03 | 4.05 | ↑ |
| 10 | Glycerol | 10.01 | 72.05 | B-10 vs H-10 | 4.94 | 0.01 | 0.37 | ↓ |
|  |  |  |  | B-21 vs H-21 | 5.29 | 0.02 | 0.54 | ↓ |
| 11 | L-Proline | 10.27 | 128.00 | B-10 vs L-10 | 3.90 | 0.03 | 0.72 | ↓ |
|  |  |  |  | B-10 vs H-10 | 1.20 | 0.01 | 0.12 | ↓ |
|  |  |  |  | B-21 vs L-21 | 1.21 | 0.03 | 0.36 | ↓ |
| 12 | beta-Alanine | 10.43 | 247.99 | B-21 vs H-21 | 1.58 | 0.02 | 2.66 | ↑ |
| 13 | Pyrimidine | 10.85 | 255.00 | B-10 vs H-10 | 1.90 | 0.03 | 2.30 | ↑ |
| 14 | L-Aspartic acid | 13.13 | 100.00 | B-10 vs L-10 | 1.35 | 0.01 | 0.58 | ↓ |
|  |  |  |  | B-10 vs H-10 | 1.24 | 0.01 | 3.43 | ↑ |
| 15 | D-Glucuronic acid | 14.26 | 245.99 | B-21 vs L-21 | 1.05 | 0.04 | 0.16 | ↓ |
| 16 | Phenylalanine | 14.41 | 191.99 | B-10 vs H-10 | 1.27 | 0.00 | 3.93 | ↑ |
| 17 | D-Lyxose | 14.84 | 59.09 | B-21 vs L-21 | 1.37 | 0.00 | 2.23 | ↑ |
|  |  |  |  | B-21 vs H-21 | 1.03 | 0.00 | 1.37 | ↑ |
| 18 | DL-Arabinose | 14.85 | 73.07 | B-10 vs L-10 | 6.15 | 0.02 | 1.91 | ↑ |
|  |  |  |  | B-10 vs H-10 | 5.84 | 0.02 | 2.56 | ↑ |
|  |  |  |  | B-21 vs L-21 | 1.02 | 0.00 | 2.43 | ↑ |
| 19 | D-Rhamnose | 15.61 | 117.00 | B-10 vs L-10 | 2.95 | 0.03 | 2.17 | ↑ |
| 20 | D-Glucose | 17.41 | 157.00 | B-10 vs H-10 | 1.45 | 0.04 | 0.25 | ↓ |
|  |  |  |  | B-21 vs H-21 | 1.19 | 0.02 | 0.27 | ↓ |
| 21 | L-Lysine | 17.45 | 127.99 | B-10 vs H-10 | 4.90 | 0.03 | 0.38 | ↓ |
|  |  |  |  | B-21 vs L-21 | 5.78 | 0.05 | 0.42 | ↓ |
|  |  |  |  | B-21 vs H-21 | 5.91 | 0.04 | 0.51 | ↓ |
| 22 | Octadecenoic acid | 19.04 | 55.09 | B-10 vs H-10 | 1.32 | 0.00 | 0.19 | ↓ |
|  |  |  |  | B-21 vs H-21 | 1.12 | 0.05 | 0.39 | ↓ |
| 23 | Inositol | 19.26 | 319.17 | B-21 vs L-21 | 1.46 | 0.04 | 0.21 | ↓ |
|  |  |  |  | B-21 vs H-21 | 1.58 | 0.04 | 0.26 | ↓ |

Note: ^a^ VIP value was obtained from OPLS-DA. ^b^ Fold change (FC) was calculated as the ratio of the average relative level between two groups (FC value=B-10/L-10 or B-10/H-10 or B-21/L-21 or B-21/H-21). ^c^ Trend: ↑ Metabolites increased in B-10 or B-21 group; ↓ Metabolites decreased in B-10 or B-21 group.

**Table S5** The differential metabolites of fecal samples at different day of the same dose groups.

| No | Metabolites | RT  (min) | Mass  (*m/z*) | group | VIP value ^a^ | *P* value | FC ^b^ | Trend |
| --- | --- | --- | --- | --- | --- | --- | --- | --- |
| 1 | Thiourea | 5.78 | 171.00 | L-0 vs L-10 | 1.78 | 0.01 | 0.34 | ↓ |
| 2 | Pentanoic acid | 5.90 | 70.09 | L-0 vs L-10 | 1.98 | 0.02 | 0.44 | ↓ |
|  |  |  |  | L-0 vs L-21 | 1.31 | 0.02 | 1.52 | ↑ |
| 3 | Butane | 6.71 | 151.95 | L-0 vs L-10 | 1.42 | 0.00 | 0.22 | ↓ |
| 4 | D-Lactic acid | 7.00 | 59.09 | L-0 vs L-10 | 1.49 | 0.01 | 1.85 | ↑ |
|  |  |  |  | L-0 vs L-21 | 1.66 | 0.01 | 1.76 | ↑ |
|  |  |  |  | H-0 vs H-10 | 2.67 | 0.04 | 1.86 | ↑ |
| 5 | Hexanoic acid | 7.06 | 75.06 | L-0 vs L-10 | 1.14 | 0.00 | 2.41 | ↑ |
| 6 | L-Valine | 7.33 | 55.09 | H-0 vs H-10 | 1.16 | 0.05 | 1.48 | ↑ |
| 7 | L-Alanine | 7.59 | 72.09 | L-0 vs L-10 | 1.06 | 0.01 | 0.39 | ↓ |
| 8 | Butanoic acid | 8.44 | 243.03 | L-0 vs L-10 | 1.28 | 0.00 | 0.13 | ↓ |
| 9 | L-Norvaline | 9.16 | 144.00 | L-0 vs L-10 | 2.38 | 0.02 | 0.26 | ↓ |
| 10 | Glycerol | 10.01 | 72.05 | L-0 vs L-10 | 2.33 | 0.03 | 2.48 | ↑ |
|  |  |  |  | L-0 vs L-21 | 2.94 | 0.02 | 3.03 | ↑ |
|  |  |  |  | H-0 vs H-10 | 6.53 | 0.00 | 0.33 | ↓ |
|  |  |  |  | H-0 vs H-21 | 0.23 | 0.00 | 9.81 | ↑ |
| 11 | L-Threonine | 10.30 | 298.08 | L-0 vs L-10 | 1.62 | 0.04 | 0.27 | ↓ |
| 12 | Butanedioic acid | 10.45 | 174.09 | L-0 vs L-21 | 1.25 | 0.02 | 0.39 | ↓ |
| 13 | Pyrimidine | 10.85 | 255.00 | L-0 vs L-10 | 1.45 | 0.03 | 0.32 | ↓ |
|  |  |  |  | L-0 vs L-21 | 1.51 | 0.05 | 0.34 | ↓ |
| 14 | L-Aspartic acid | 13.13 | 100.00 | L-0 vs L-10 | 1.38 | 0.00 | 0.30 | ↓ |
|  |  |  |  | L-0 vs L-21 | 1.24 | 0.00 | 0.36 | ↓ |
|  |  |  |  | H-0 vs H-10 | 1.06 | 0.03 | 0.52 | ↓ |
| 15 | Aminohexanoic acid | 14.26 | 245.99 | L-0 vs L-21 | 1.19 | 0.00 | 0.11 | ↓ |
| 16 | Benzeneacetic acid | 14.40 | 192.00 | L-0 vs L-21 | 1.03 | 0.03 | 0.25 | ↓ |
|  |  |  |  | H-0 vs H-21 | 1.09 | 0.00 | 12.43 | ↑ |
| 17 | Phenylalanine | 14.41 | 191.99 | L-0 vs L-10 | 3.38 | 0.00 | 0.05 | ↓ |
|  |  |  |  | L-0 vs L-21 | 1.87 | 0.01 | 0.14 | ↓ |
| 18 | DL-Arabinose | 14.87 | 73.07 | L-0 vs L-10 | 4.04 | 0.01 | 2.52 | ↑ |
|  |  |  |  | L-0 vs L-21 | 4.78 | 0.00 | 2.60 | ↑ |
|  |  |  |  | H-0 vs H-10 | 3.48 | 0.04 | 1.42 | ↑ |
| 19 | D-Rhamnose | 15.61 | 117.00 | L-0 vs L-10 | 3.75 | 0.00 | 3.50 | ↑ |
|  |  |  |  | L-0 vs L-21 | 3.62 | 0.00 | 2.35 | ↑ |
|  |  |  |  | H-0 vs H-21 | 4.47 | 0.03 | 2.48 | ↑ |
| 20 | D-Mannose | 17.39 | 147.00 | H-0 vs H-21 | 1.87 | 0.03 | 1.55 | ↑ |
| 21 | L-Lysine | 17.45 | 127.99 | L-0 vs L-21 | 1.96 | 0.00 | 0.47 | ↓ |
| 22 | d-Galactose | 17.52 | 232.99 | H-0 vs H-21 | 1.02 | 0.03 | 2.22 | ↑ |
| 23 | Galactaric acid | 17.90 | 332.99 | H-0 vs H-21 | 2.02 | 0.04 | 10.65 | ↑ |
| 24 | Glucopyranose | 18.12 | 203.99 | H-0 vs H-21 | 2.07 | 0.04 | 24.05 | ↑ |
| 25 | Octadecenoic acid | 19.04 | 55.09 | H-0 vs H-10 | 1.03 | 0.03 | 0.59 | ↓ |
| 26 | N-Acetyl-D-glucosamine | 19.18 | 69.05 | H-0 vs H-10 | 1.13 | 0.04 | 0.37 | ↓ |
| 27 | Inositol | 19.26 | 319.17 | H-0 vs H-10 | 1.60 | 0.04 | 0.52 | ↓ |
| 28 | Isoquinoline | 23.20 | 191.99 | H-0 vs H-10 | 1.24 | 0.01 | 36.58 | ↑ |
|  |  |  |  | H-0 vs H-21 | 1.28 | 0.03 | 29.47 | ↑ |

Note: ^a^ VIP value was obtained from OPLS-DA. ^b^ Fold change (FC) was calculated as the ratio of the average relative level between two groups (FC value=L-0/L-10 or L-0/L-21 or H-0/H-10 or H-0/H-21). ^c^ Trend: ↑ Metabolites increased in L-0 or H-0 group; ↓ Metabolites decreased in L-0 or H-0 group.
